# Two-Component MprAB System Regulates the Expression of Genes Involved in Cell Envelope Biosynthesis in *Corynebacterium glutamicum*

**DOI:** 10.3390/microorganisms13051120

**Published:** 2025-05-13

**Authors:** Yu Zou, Danni Huang, Xiuxia Liu, Yankun Yang, Chunli Liu, Ye Li, Zhonghu Bai

**Affiliations:** 1 School of Biotechnology and Key Laboratory of Industrial Biotechnology of Ministry of Education, Jiangnan University, Wuxi 214122, China; jndxzy1997@163.com (Y.Z.); 6200203003@stu.jiangnan.edu.cn (D.H.); yangyankun@jiangnan.edu.cn (Y.Y.); liuchunli@jiangnan.edu.cn (C.L.); liye@jiangnan.edu.cn (Y.L.); 2 National Engineering Research Center for Cereal Fermentation and Food Biomanufacturing, Jiangnan University, Wuxi 214122, China; 3 Jiangsu Provincial Engineering Research Center for Bioactive Product Processing, Jiangnan University, Wuxi 214122, China; 4 Zhengzhou University of Technology, Zhengzhou 450044, China

**Keywords:** *C. glutamicum*, two-component system, MprAB, cell envelope, HtrA, L-alanine

## Abstract

To accurately detect internal and environmental cues, bacteria have evolved signal transduction pathways such as two-component systems (TCSs) to reprogram appropriate genetic and physiological functions for adaptation and survival. The MprAB TCS is commonly found in actinobacteria and has been associated with important processes such as mycobacterial virulence, nutrient starvation, and environmental stress, particularly cell envelope stress. However, a comprehensive investigation of the function and response network of the MprAB TCS in corynebacteria remains to be carried out. In this study, we report that the MprAB TCS (previously named CgtSR2) plays a critical role in regulating genes involved in cell envelope remodeling in *C. glutamicum*. The results indicated that the MprAB TCS directly controls a broad regulon, including cell wall biosynthesis proteins, alternative sigma factors, secreted proteins of unknown function, and the *mprAB* gene locus itself. Among these, the HtrA-like serine protease confers vancomycin and penicillin resistance. Furthermore, we found that the function of the cell envelope was disrupted during overexpression of *mprA*, resulting in elongated cell morphology and increased cell membrane permeability, as well as enhanced excretion of L-alanine. In conclusion, our findings provide novel insights into how the conserved MprAB TCS controls cell envelope homeostasis in distant actinobacteria.

## 1. Introduction

*Corynebacterium glutamicum* is a Gram-positive bacterium belonging to the soil Actinobacteria, which share an atypical cell envelope structure with the pathogen*s Corynebacterium diphtheriae* and *Mycobacteria tuberculosis* [1,2]. These organisms have a cytoplasmic membrane surrounded by an indispensable cell wall matrix consisting of a peptidoglycan polymer, a polysaccharide layer consisting of arabinogalactans, and an outer membrane layer of long-chain fatty acids called mycolic acids [3]. The cell envelope architecture of these bacteria is critical both for resisting environmental stress and enabling biomolecular transport [4,5].

Widespread in prokaryotes, two-component systems (TCSs) are important signal transduction pathways by which bacteria reprogram their physiology to adapt to environmental cues [6,7,8]. Prototypical TCSs consist of a histidine kinase (HK) with a transmembrane domain and a cognate response regulator (RR). In response to specific external stimuli, including pH, temperature, osmolarity, and antibiotics, HK autophosphorylates conserved histidine residues and transfers a phosphate group to a conserved aspartate residue of RR, which regulates gene expression [9,10,11]. Many TCSs respond to signals by regulating the function of the cell envelope, such as MtrAB and MprAB TCS [12,13].

The MprAB TCS consists of MprA (RR) and MprB (HK), a highly conserved two-component system in Actinobacteria. In mycobacteria, the MprAB TCS is involved in the response to environmental stress, particularly its effect on the cell envelope [14]. For example, transcription of the operon encoding MprAB TCS in *Mycobacterium tuberculosis* can be induced by cell wall stress during infection, low concentrations of surfactants (sodium dodecyl sulfate or Triton X-100), and nutrient starvation [15,16,17]. The MprAB TCS and alternative sigma factor sigE (σ^E^) are at the cornerstone of a complex genetic network that responds to cell envelope stress. The MprAB TCS regulates more than 200 genes involved in cell wall homeostasis and secretion of the ESX-1 system in *M. tuberculosis*, such as *sigE*, *sigB*, *clgR*, *htrA*, *pspA*, *acr2*, *espA*, *espR*, and *rel* [18,19,20,21,22,23,24,25,26]. The *M. tuberculosis* ESX-1 (type VII secretion systems) system mediates innate mechanisms against pathogens [27]. MprAB TCS deletion leads to hypervirulence of *M. tuberculosis*, whereas the *M. bovis* BCG deletion strain is attenuated in macrophages [28]. In *Streptomyces albulus*, the MprAB TCS is upregulated by pH shock during ε-poly-L-lysine production [29]. Thirteen TCSs have been identified in the *C. glutamicum* genome, with two TCSs functionally characterized in regulating cell envelope homeostasis [30,31]. These findings elucidate the critical role of TCS in regulating the cell envelope stress response. CgtSR2 is homologous to the MprAB TCS in *Mycobacterium* spp. However, its target genes, as well as the signals recognized by MprB, are completely unknown. In this study, the function and regulatory network of the MprAB TCS were investigated using *C. glutamicum* as a model organism. We investigated the role of the MprAB TCS in regulating cell envelope biosynthesis in *C. glutamicum* through gene expression profiling and phenotypic analysis. Transcriptomic and electrophoretic mobility shift assay (EMSA) analyses revealed direct binding of MprA to the promoter regions of cell envelope-associated genes, including *sigB* and *sigE*. Furthermore, overexpression of *mprA* significantly enhanced L-alanine production, suggesting that remodeling cell envelope architecture by activating MprA could effectively promote amino acid excretion. Finally, we explored the response of the MprAB TCS and its regulated genes, such as *htrA*, to the cell envelope stressors vancomycin and penicillin. Overall, our results indicate that the MprAB TCS of *C. glutamicum* represents a novel regulatory mechanism for actinobacteria to adapt to cell envelope stress.

## 2. Materials and Methods

Strains, plasmids, and cultivation. The strains and plasmids used in this study are listed in Table S1. *C. glutamicum* strains were grown in Luria–Bertani broth (LBB) medium (LB medium supplemented with brain heart infusion broth), LBHIS medium (LBB medium with 91 g/L sorbitol), and CGXII [32] medium containing 4% (*w*/*v*) glucose at 30 °C. *E. coli* strains were cultured in LB medium (tryptone, 10 g/L, yeast extract, 5 g/L, NaCl 10 g/L) at 37 °C. When necessary, 50 μg/mL kanamycin was used for both *C. glutamicum* and *E. coli*, 30 μg/mL chloramphenicol was used for *E. coli*, and 10 μg/mL was used for *C. glutamicum*.

Plasmid construction. The primers used are listed in Table S2. The primers were designed using the NCBI database. For MprA overexpression and purification, *mprA* was cloned by PCR with the primers OPmprA-F and OPmprA-R using genomic DNA from *C. glutamicum* ATCC 13032 as a template. The plasmid pET28a (Novagen, Beijing, China) backbone was amplified using the primers pET28a-F and pET28a-R. The two PCR fragments were assembled seamlessly (ABclonal, Wuhan, China) into the plasmid pET-*mprA*-CHis_6_. The primers OpmprA_D-N_-F and OpmprA_D-N_-R with the primers OpmprA_D-E_-F and OpmprA_D-E_-R were used for site-directed mutagenesis, resulting in pET-*mprA_D-N_*-CHis_6_ and pET-*mprA_D-E_*-CHis_6_.

To overproduce MprA and HtrA in *C. glutamicum*, *mprA* and *htrA* were cloned by PCR with the primers OPmprA-F and OPmprA-R and ORhtrA-F and ORhtrA-R using genomic DNA from *C. glutamicum* ATCC 13032 as a template. The IPTG-inducible pXMJ19 plasmid backbone was amplified using the primers pXMJ19-F and pXMJ19-R. The two PCR fragments were seamlessly assembled into the plasmids pXMJ19-*mprA* and pXMJ19-*htrA*.

To monitor promoter activity by fluorescence characterization, the regions approximately 250 bp upstream and 60 bp downstream of the start codon of *mprA* (P*_mprA_*-F and P*_mprA_*-R) and 400 bp upstream and 75 bp downstream of the start codon of *htrA* were cloned by PCR using genomic DNA from *C. glutamicum* ATCC 13032 as a template. A biscistronic element containing another ribosome-binding site was added, and a stop codon was inserted in front of the start codon of an *egfp* reporter. The promoterless plasmid pECX1 (promoterless-EGFP-terminator-H_36_-mCherry) was amplified using the primers pECX1-F and pECX1-R. The two PCR fragments were seamlessly assembled into pECX1-P*_mprA_*and pECX1-P*_htrA_*.

Strain construction. For gene deletion in *C. glutamicum*, we used the suicide plasmid pK18mobsacB via double homologous recombination. Approximately 1000 bp of the homologous upstream and downstream sequences of *mprA* were amplified with the primers KomprA-F1 and KomprA-R1 and the primers KomprA-F2 and KomprA-R2 using genomic DNA from *C. glutamicum* ATCC 13032 as a template. Both PCR fragments were seamlessly assembled into *Hind III*/*EcoRI*–digested pK18mobsacB, which was electroporated into *C. glutamicum* ATCC13032. We used LBHIS agar plates with 25 μg/mL kanamycin to select the transformants which had integrated the plasmid into the genomic DNA of *C. glutamicum* ATCC 13032. The resulting Kan^R^ colonies were grown in LBB liquid medium at 30 °C overnight. The culture was spread on an LBHIS agar plate with 10% sucrose, leading to the loss of *sacB*. Deletion of *mprB* in the △*mprA* mutant background was performed using the same procedure with the primers KomprA-F1, KomprB-R1, and KomprB-F2. Deletion of *htrA* in the wild-type background was performed with the primers KohtrA-F1, KohtrA-R1, and KohtrA-F2. Deletion of *mprA*, *mprAB,* and *htrA* was confirmed by colony PCR using the primer pairs cxmprA-F and cxmprA-R, cxmprAB-F and cxmprAB-R, and cx-htrA-F and cx-htrA-R.

To complement the △*htrA* mutant, *htrA* was amplified by PCR using genomic DNA from *C. glutamicum* ATCC 13032 and the primers ChtrA-F and ChtrA-R. The pXMJ19 plasmid backbone was amplified using the primers CpXMJ19-F and pXMJ19-R. Subsequently, the two PCR fragments were seamlessly assembled into the plasmids.

Global transcriptome analysis. To compare the wild-type and WT △*mprA* mutant strains, a single clone was grown in LBB medium at 30 °C for 16 h. The pre-culture was transferred into CGXII medium containing 4% glucose at an initial OD_600_ = 0.5 and then harvested at OD_600_ = 5.0–6.0. To compare MprA expression from the plasmid in the WT △*mprA* mutant strain and the empty plasmid in the WT △*mprA* mutant strain, 1 mM IPTG was added to the medium at OD_600_ = 5.0–6.0. The cultures were harvested after 1 h. The cells were washed three times using PBS buffer (137 mM NaCl, 2.7 mM KCl, 4.3 mM Na_2_HPO_4_, 1.4 mM KH_2_PO_4_) and harvested by centrifugation at 4 °C, and the pelleted cells were quick-frozen in liquid nitrogen. RNA-seq was performed by Novogene (Beijing, China). Comparisons were performed using three biological replicates. Sequencing adapters and low-quality reads were removed using Fast QC software 0.11.5. High-quality RNA-seq data were assembled and aligned using annotated reference DNA sequence data (GCA_002847405.1) and RNASTAR 2.7.11. Differentially expressed genes were identified using DESeq2 (|fold change| ≥ 2, *p* < 0.05). The raw reads from the RNA-seq analysis have also been uploaded to the SRA database and are available under accession number PRJNA1218008.

Next, 1 mL mid-log phase *C. glutamicum* cultures from 24 deep-well plates was harvested and rapidly frozen in liquid nitrogen. Total RNA was extracted using an RNApure Bacteria Kit (Cwbio, Beijing, China), and cDNA was synthesized from the mRNA with HiScript III RT SuperMix (Vazyme, Nanjing, China). qPCR reactions were prepared with ChamQ SYBR Master Mix (Vazyme, Nanjing, China), 0.4 μM primers (Table S2), and 1 μL cDNA and run on a StepOne Plus system (ABI, Waltham, USA) under the following cycling conditions: 95 °C (1 min); 40 cycles of 95 °C (15 s), 55 °C (15 s), 72 °C (20 s). Relative gene expression (2^(−ΔΔCt)^) was normalized to 16S rRNA and analyzed using Excel 2020.

Overproduction and purification of MprA, MprA(D51N), and MprA(D51E). *E. coli* BL21 (DE3) containing plasmid pET28a derivatives with MprA, MprA (D51N), and MprA (D51E) was grown in TB medium (tryptone, 12 g/L; yeast extract, 24 g/L, glycerol, 4 mL/L, K_2_HPO_4_, 12.54 g/L, KH_2_PO_4_, 2.31 g/L) at 37 °C overnight. Cultures were diluted 1:100 and grown in 250 mL of fresh TB medium until the OD_600_ reached 0.4–0.6. Next, 1 mM IPTG was added to the culture medium for 24 h at 16 °C to induce protein expression. Next, the cells were harvested by centrifugation at 4000× *g* for 30 min at 4 °C, and the pelleted cells were resuspended in 50 mL Buffer A (20 mM Tris, 500 mM NaCl, 10 mM imidazole, 5% glycerol, pH 7.5) supplemented with a protease and phosphatase inhibitor cocktail (Beyotime, Haimen, China). The cells were disrupted by high-pressure homogenization, followed by centrifugation at 4000× *g* for 30 min at 4 °C. His-tagged MprA, MprA (D51N), and MprA (D51E) proteins present in the supernatant were purified by affinity chromatography using PrePack Ni-NTA agarose (Yesean, Shanghai, China) in an AKTA purifier system (GE, Boston, USA). The proteins were washed with Buffers A and B (20 mM Tris, 500 mM NaCl, 50 mM imidazole, 5% glycerol), eluted with Buffer C (20 mM Tris, 500 mM NaCl, 200 mM imidazole, 5% glycerol), and desalted into Buffer P (50 mM Tris-HCl, 50 mM KCl, 10 mM MgCl_2_, 0.5 mM EDTA, 10% glycerol, pH 7.5). The concentrated proteins (10 kDa cutoff, Millipore (Sigma, Shanghai, China)) were quantified by BCA assay (Yeasen, Shanghai, China) and assessed for purity via SDS-PAGE.

Electrophoretic mobility shift assay. For in vitro phosphorylation reactions, purified MprA protein was incubated with 50 mM acetylphosphate in Buffer P at 30 °C for 1 h. Unphosphorylated proteins were used as a negative control. For the binding reactions, approximately 40 ng of 264–301 bp DNA fragments, 30 ng of 100 bp DNA fragments, or 20 ng of 30 bp DNA fragments (25 nM–107 nM) was mixed with 0–64-fold molar excess (0–6.9 mM) of phosphorylated MprA, unphosphorylated MprA, phosphorylation-mimic MprA (D51E), or phosphorylation-defective MprA (D51N) in a total volume of 10 μL. The protein and DNA fragments were dissolved in Buffer P. The mixture was incubated at 20 °C for 30 min and loaded onto a 6% native polyacrylamide gel. Electrophoresis was performed at 4 °C and 120 V for 1 h using cooled 0.5× TBE (89 mM Tris base, 89 mM boric acid, 2 mM Na_2_EDTA) as the buffer. Finally, the gels were stained with Safe-Red (Abclonal) to visualize the positions of the DNA fragments.

Light and fluorescence microscopy. For light microscopy, the pre-cultures were grown in CGXII medium containing 4% glucose for 24 h at 30 °C. *C. glutamicum* cells were placed on a 0.5% agar plate and analyzed microscopically using an Olympus BX53F (Olympus Europa, Hamburg, Germany) with a 100× oil immersion lens. For fluorescence microscopy, the cells were washed three times with PBS buffer and centrifuged at 4000× *g* for 2 min at 4 °C. The cell precipitate was resuspended in 500 μL PBS containing 200 ng/mL Hoechst 33342 and 300 ng/mL Nile red or 2 μg/mL PI to OD_600_ = 2.0 (Yeasen). The cells were incubated for 30 min at 37 °C in the dark. Lipids were stained with Nile red, DNA with Hoechst 33342, and dead cells with PI. The images were processed, and cell length was measured using ImageJ 1.54p.

Field emission scanning electron microscopy. For FSEM, the bacteria were fixed with 2.5% glutaraldehyde (pH 7.4) in PBS for at least 4 h, washed in 0.1 M phosphate buffer (pH 7.2) for 15 min, and dehydrated by consecutive incubation in an ascending acetone series (30%, 50%, 70%, 90%, and 100%) for 10 min each; the last step was repeated three times. The samples were critical-point-dried in liquid CO_2_ and sputter-coated with a 10 nm gold/palladium layer. The samples were analyzed using a field-emission scanning electron microscope (SU8200, Hitachi, Tokyo, Japan) with a 3 kV acceleration voltage in a high-vacuum environment.

Transmission electron microscopy. For TEM, the samples were fixed in 2.5% glutaraldehyde (pH 7.4) for 2 h and embedded in agarose with a low melting point. After washing three times with 0.1 M phosphate buffer (pH 7.2) and fixing in 1% osmic acid at 4 °C for 2 h, the samples were gradient-dehydrated using a graded series of ethanol solutions. Subsequently, the samples were embedded in Epon–Araldite resin for penetration and placed in a mold for polymerization. Ultrathin sections (UC7; Leica, Wetzlar, Germany) were collected for microstructural analysis. Counterstaining was performed with 3% uranyl acetate and 2.7% lead citrate, and the samples were observed by transmission electron microscopy (HT7700; Hitachi).

Miscellaneous procedures. For the serial dilution spot experiments, the pre-culture was cultivated in CGXII medium containing 4% glucose at an initial OD_600_ = 0.5, and the cells were grown for 4 h at 30 °C. Then, the cells were harvested by centrifugation at 4000× *g* at 4 °C, and the pellets were washed three times with PBS buffer. All cultures were normalized to OD_600_ = 1.0. Next, 2 μL of diluted culture was spotted onto LBB agar plates containing 0.25 μg/mL vancomycin or 0.2 U/mL penicillin and incubated for 36 h at 30 °C.

For fluorescence assays, *C. glutamicum* was transformed with the reporter plasmid pEC-X1-P_mprA_ or pEC-X1-P_htrA_ (Table S2). LBB medium was inoculated with a single clone from a fresh LBB agar plate and incubated for 12–16 h at 30 °C. Next, the pre-culture was transferred into fresh LBB medium at an initial OD_600_ = 0.2 and grown for 4 h at 30 °C. The samples were collected as required for measurement using a TECAN Spark microplate reader (Molecular Devices, Salzburg, Austria). EGFP (excitation wavelength, 485 nm; emission wavelength, 535 nm; signal gain factor, 144) and mCherry fluorescence (excitation wavelength, 570 nm; emission wavelength, 615 nm; signal gain factor, 145) were quantified in 96-well plates. To calculate the relative fluorescence, the net fluorescence value was measured after subtracting the background fluorescence of the strain without fluorescent protein expression.

$$Relative\;fluorescence=\frac{net\;EGFP\;fluorescence}{net\;mCheery\;fluorescence}$$

For batch fermentation, *C. glutamicum* was incubated in 5 mL of seed medium in a centrifuge tube at 30 °C. The seed medium contains 30 g/L corn steep liquor, 25 g/L glucose, 1 g/L KH_2_PO_4_, 1.25 g/L urea, 500 mg/L (NH4)_2_SO_4_, and 500 mg/L MgSO_4_. Then, the seed culture was inoculated into 50 mL fermentation medium with a starting optical density of OD_600_ = 1.0 and incubated at 30 °C for 72 h. The fermentation medium contains 100 g/L glucose, 40 g/L (NH4)_2_SO_4_, 15 g/L corn steep liquor, 1 g/L KH_2_PO_4_, 10 mg/L FeSO_4_∙7 H_2_O, 500 mg/L MgSO_4_, 10 mg/L MnSO_4_·H_2_O, 1 g/L yeast extract, 1 mg/L thiamine·HCl, and 20 g/L CaCO_3_. *mprA* overexpression was induced by supplementing the culture with 0.01 M IPTG. Samples were taken at 12 h intervals for analysis of glucose, amino acids, and OD_600_. Glucose concentration was measured using an SBA-40C bioanalyzer. The concentration of amino acids was determined using the PITC derivatization method [33]. Amino acids were analyzed via HPLC (LC-20AT, Shimadzu, Kyoto, Japan) with an Agilent HC-C18 column (4.6 × 250 mm, Agilent Technologies, Santa Clara, CA, USA) equipped with a UV detector (SPD-20A, Shimadzu, Kyoto, Japan) at 254 nm. The column temperature was operated at 40 °C, and the mobile phase was a mixture of A (0.1 M CH_3_COONa, pH 6.5) and B (80% acetonitrile) at a flow rate of 1 mL/min.

## 3. Results

### 3.1. The MprAB Two-Component System Is Conserved in Corynebacterium and Mycobacterium

**Figure 1:**
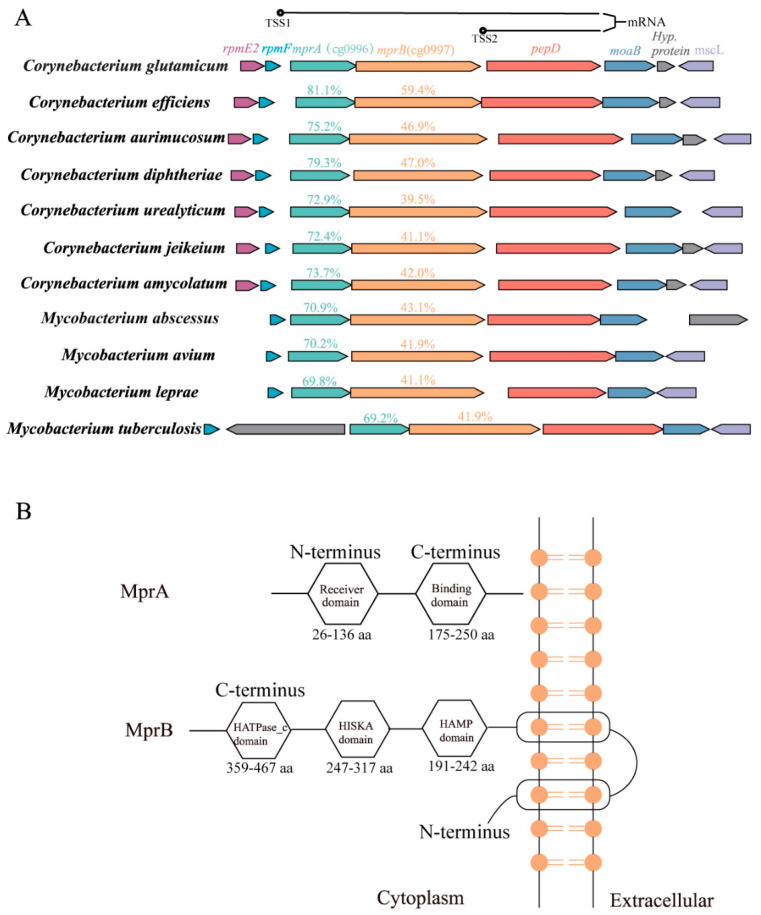
The MprAB two-component system. (**A**) Genomic organization of the mprAB locus in *Corynebacterium* and *Mycobacterium*. Amino acid sequence identity with the *C. glutamicum* MprA and MprB ortholog is given in the upper panel. TSS: transcriptional start site. The genomic context of MprAB TCS was extracted from MicrobesOnline (http://microbesonline.org) (accessed on 1 October 2024). (**B**) Prediction of the protein domains of MprA and MprB, extracted from KEGG.

The MprAB (cgtS2/R2) system is considered a potential two-component system in *C. glutamicum.* However, its specific function in *C. glutamicum* has not been reported. While MprA is highly conserved among both *Corynebacterium* and *Mycobacterium*, MprB displays greater variability among different species, including prominent pathogens like *C. diphtheriae* (with 46.98% sequence identity) and *M. tuberculosis* (with 41.85% sequence identity) (Figure 1A). The regulator, MprA, contains putative N-terminal receiver (26–133 aa) and C-terminal DNA-binding (175–250 aa) domains. The sensor kinase MprB possesses two transmembrane helices flanking the cytoplasmic HAMP domain (191–242 aa), followed by the HisKA (247–317 aa) and HATPase_c domains (359–467 aa) (Figure 1B). In mycobacteria, the MprAB TCS is part of the regulatory network for responding to cell envelope stress [14]. Owing to its phylogenetic conservation, the MprAB TCS may also be important in the response to cell envelope stress in *C. glutamicum*.

### 3.2. Transcriptome Profile of Altered mprA Expression

To identify the genes regulated by *mprA* genome-wide, we performed a comparative transcriptome analysis of *C. glutamicum* wild-type vs. △*mprA*, and of the △*mprA* mutant strain containing the empty plasmid pXMJ19 vs. overexpressing *mprA* from plasmid pXMJ19-*mprA* in the △*mprA* mutant strain, by RNA-seq. We also selected several samples and verified the accuracy of the transcriptome using RT-qPCR (Figure S1). RNA-seq analysis revealed no significant differences in the transcription levels of *sigE* and *sigB* upon overexpressing *mprA*. However, the qRT-PCR results indicated that their transcriptional abundance was downregulated in the △*mprA*/pXMJ19-*mprA* mutant (Figure S1). We propose that the primary inconsistencies between the qRT-PCR and RNA-seq results arise from the lower sensitivity of RNA-seq and variations in statistical methods [34,35]. Furthermore, it is worth noting that these genes are directly regulated by *mprA* in mycobacteria. Altogether, RNA-seq revealed that, in three biologically independent replicate experiments, 12 genes were significantly (*p* < 0.05) upregulated by more than two-fold, and 14 genes were significantly downregulated by more than two-fold in the △*mprA* mutant compared to the wild-type strain. Overexpression of *mprA* had an obvious global transcriptome impact in the △*mprA* mutant strain. In total, 131 genes were differentially expressed.

Under the test conditions, many differentially expressed genes were involved in cell envelope biosynthesis (Table 1). Bla, a member of the penicillin-binding protein family, elongates glycan strands and cross-links peptide stems. WZZ participates in cell surface polysaccharide biosynthesis. L, D-transpeptidases such as Cg0650 play a role in cross-link formation within peptidoglycans. The LGFP domains of Csp, Cg2069, and Psp2 stabilize the outer membrane by interacting with the peptidoglycan layer. Additionally, Cmt1, PorA, and PorB are involved in mycolate synthesis [36,37,38,39]. All of these genes were repressed by *mprA* overexpression, suggesting the importance of MprAB TCS in regulating cell envelope remodeling. These findings support the hypothesis that *mprA* overexpression inhibits synthesis of the cell envelope, resulting in significantly elongated morphology compared with the wild-type strain.

Notably, hydrolase *cg0793* and the zinc metallochaperone *ycic* exhibit strong *mprA* dependence. Both were activated in the Zn^2+^ uptake regulator *zur* mutant strain [40]. Additionally, deletion of *mprA* resulted in reduced mRNA levels of the *znr-zur* operon, which is involved in zinc homeostasis. Overall, transcriptomic and qRT-PCR studies show that MprAB is predominantly a repressor that regulates the transcript levels of the cell envelope-related genes *sigE* and *sigB* in *C. glutamicum*, unlike its function in mycobacteria [26].

### 3.3. Identification of mprA-Binding Regions

**Figure 2:**
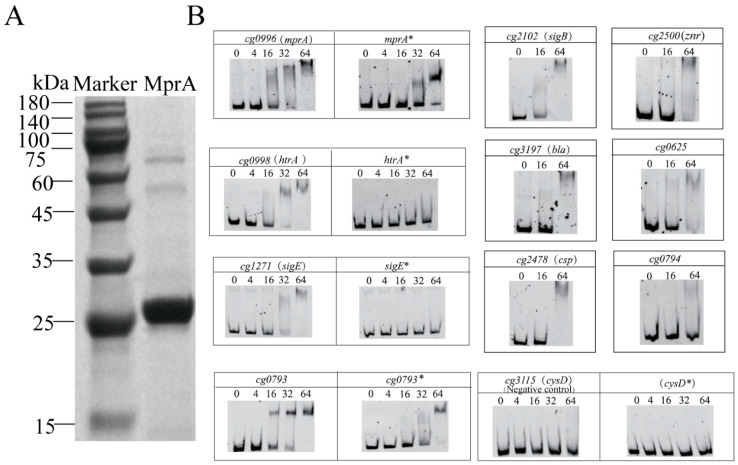
Electrophoretic mobility shift assays (EMSAs) were performed to identify binding of MprA to promoter regions. (**A**) SDS-PAGE of the MprA protein. (**B**) DNA fragments about 300 bp upstream of the putative MprA target genes were incubated with or without a 4, 16, 32, or 64 molar excesses of phosphorylated purified MprA protein, as indicated below the respective lanes. The promoter region of the untargeted gene *cysD* was used as a negative control. The asterisk (*) indicates samples without prior phosphorylation of MprA by acetylphosphate. No DNA binding was observed without previous phosphorylation of MprA with acetylphosphate (htrA*, sigE*, and cysD*).

To investigate the genes directly targeted by MprA, we conducted electrophoretic mobility shift assays (EMSAs). Purified phosphorylated MprA pre-incubated with the high-energy phosphate donor acetylphosphate and unphosphorylated MprA, phosphorylation-defective MprA (D51N), and phosphorylation-mimicking MprA (D51E) were individually used for experimentation (Figure 2A and Figure S2A,B). Surprisingly, concentration-dependent binding of both MprA variants to the promoter regions of *mprA* and *cg0793* was observed (Figure 2). However, phosphorylated MprA showed significant band shifts at lower protein concentrations, suggesting that phosphorylation is not absolutely required for binding these promoter regions in vitro but can enhance its affinity for dsDNA. Furthermore, phosphorylated MprA binds to the upstream sequences of *sigE* and *htrA*, whereas unphosphorylated MprA fails to do so (Figure 2). This suggests that phosphorylation is likely indispensable for regulators to interact with dsDNA in vitro for certain genes. Likely, phosphorylation-defective MprA fails to bind to the promoter regions of *sigE* and *htrA* (Figure S2C). However, phosphorylation-mimicking MprA also binds to the promoter sequences of *mprA*, *cg0793*, *sigE,* and *htrA* (Figure S2C). The differential binding affinity of MprA protein toward these promoter sequences could be attributed to divergence in the conservation of their DNA motif sequences. Phosphorylated MprA also binds to the promoter regions of the cell envelope biosynthesis genes *bla* and *csp* and significantly binds to the upstream sequences of *cg0625* (secreted proteins of unknown function), *znr*, and *sigB* (Figure 2). Moreover, phosphorylated and unphosphorylated MprA failed to bind to the upstream promoter regions of the potential target genes *ycic* and the negative control *cysD* (Figure 2). Altogether, MprAB directly regulates the stress response–related sigma factors *sigE* and *sigB* and a range of genes involved in cell envelope biosynthesis (*bla*, *csp*, and *htrA*), strongly emphasizing its importance in regulating cell wall synthesis.

**Figure 3:**
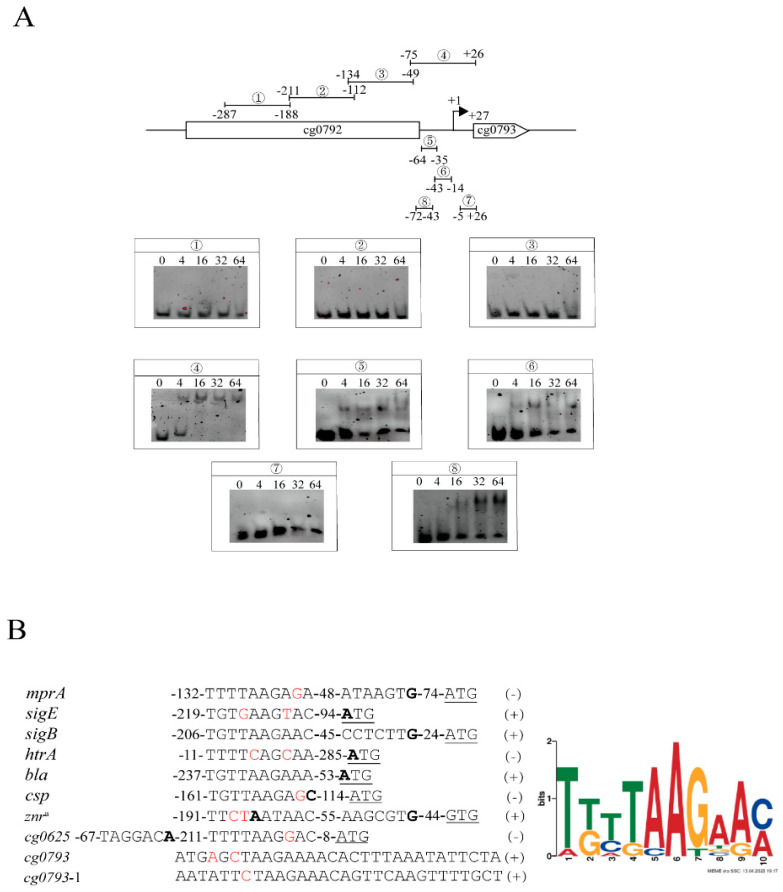
Identification of the MprA-binding motif using MEME 5.5.7 software. (**A**) Search for MprA binding sites within the promoter region of *cg0793*. (**B**) MprA consensus binding motif predicted by MEME 5.5.7 software based on analysis of the upstream sequence of verified MprA-targeted genes (left panel). The TSSs are indicated in bold and were determined by 5′-end RNA-seq [41]. The start codons are underlined. Mismatched bases in the conserved motif are shown in red. The TSS of *znr* was derived from Schroder’s study [42]. Binding of MprA to the coding strand (+) or template strand (−) in the EMSA experiments.

To identify a common motif, truncated fragments (labels 1–8) of the promoter region of *cg0793* were tested due to its high affinity for MprA. As shown in Figure 3A, a significant gel shift with 100 bp and 30 bp fragments was observed at a four-fold molar excess of the phosphorylated MprA protein (labels 4–6, 8), suggesting the presence of two MprA binding sites in the upstream sequence of *cg0793* between −64 and −35 and between −43 and −14 (labels 5 and 6). The 10 MprA-targeted gene binding sites were analyzed via EMSA experiments using MEME 5.5.7 software. The results revealed a 10 bp common motif (consensus sequence: TKTTAAGAAM; K: G/T; M: A/C) that tolerated mismatches in conserved positions, indicating that the MprA-binding motif represents a loose sequence (Figure 3B).

### 3.4. Overexpression of mprA Caused Cell Envelope Defects and Increased Alanine Titers in C. glutamicum

**Figure 4:**
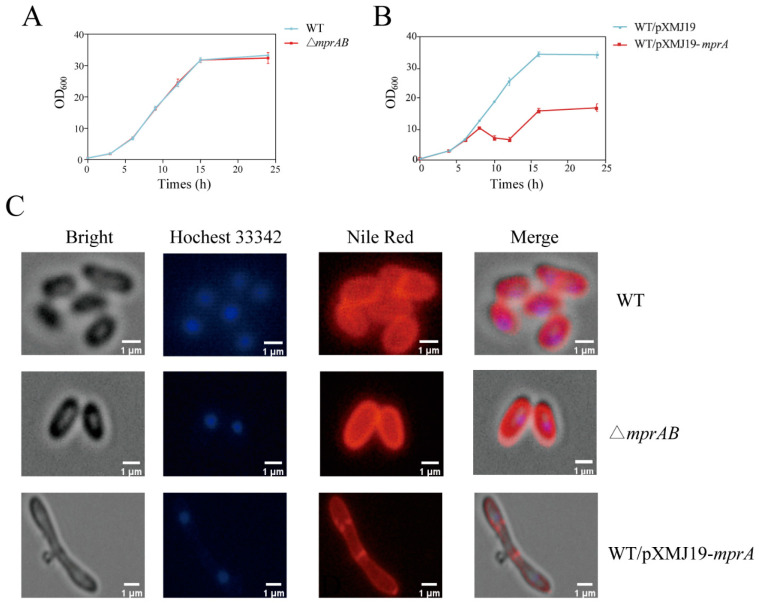
Characterization of wild-type (WT), △*mprAB* mutant, and *mprA-*overexpressing strains. Pre-cultured cells were cultivated in 3 mL of LBB medium in a 50 mL centrifuge tube at 30 °C overnight. (**A**) Growth curves of WT and △*mprAB* mutant strains in the CGXII medium containing 4% (*w*/*v*) glucose. (**B**) Growth curves of the WT strain carrying the empty plasmid pXMJ19 and pXMJ19-mprA in CGXII medium containing 4% glucose with 1 mM IPTG. (**C**) Fluorescence microscopy of the WT, △*mprAB* mutant, and *mprA-*overexpressing strains grown in CGXII medium containing 4% glucose for 24 h. The expression of *mprA* is induced by the addition of 1 mM IPTG. The lipid components of the cell membrane and DNA were marked using Nile red (red) and Hoechst 33342 (blue), respectively. The data are presented as mean ± SD of three independent biological replicates.

To characterize the physiological function of *mprA* in *C. glutamicum*, we compared the wild-type, △*mprAB* mutant, and WT/pXMJ19-*mprA* strains. The growth of the △*mprAB* mutant strain was similar to that of the wild-type strain in CGXII medium with 4% glucose at 30 °C (Figure 4A). However, *mprA* overexpression resulted in pronounced growth inhibition, and a much lower cell optical density was observed in the stationary phase (OD_600_, 33.29 ± 0.29 for WT/pXMJ19 strain versus 17.04 ± 0.60 for WT /pXMJ19-*mprA* strain) (Figure 4B).

**Figure 5:**
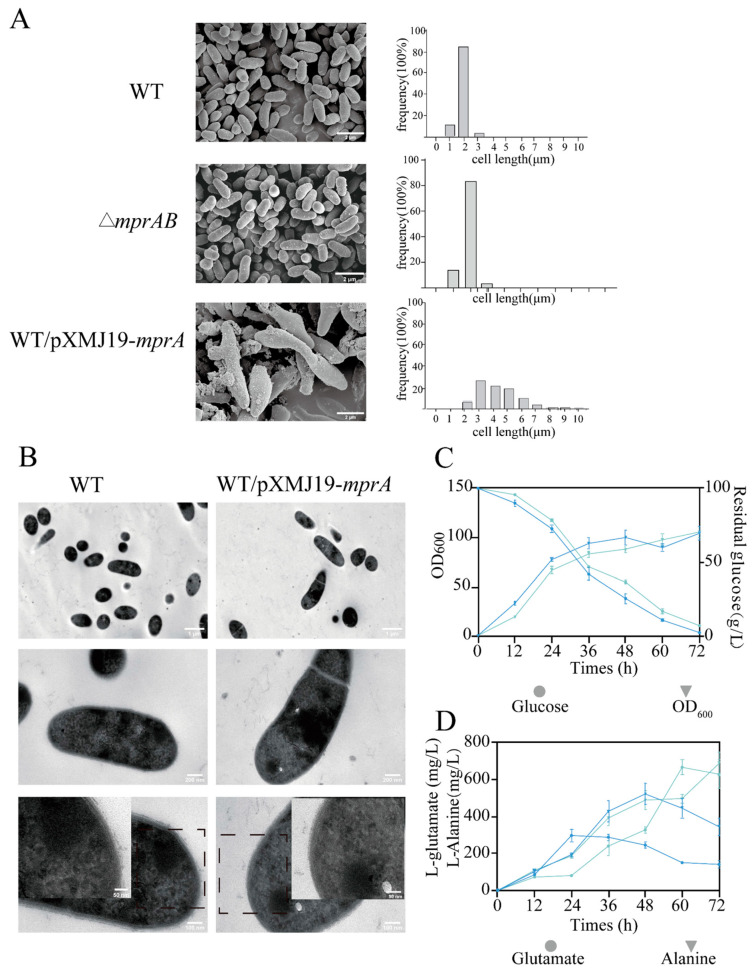
Physiological characteristics of *C. glutamicum* cells. For microscopic analysis, cells were cultured in CGXII minimal medium containing 4% glucose for 24 h, and *mprA* expression was induced by adding 1 mM IPTG. (**A**) FSEM micrographs of WT, △*mprAB* mutant, and *mprA*-overexpressing strains. FSEM pictures were captured at 10,000× magnification. Size distribution of WT, △*mprAB* mutant, and *mprA-*overexpressing strains. The lengths of at least 100 individual cells were measured. Cells were fixed using 3% glutaraldehyde. (**B**) TEM images of wild-type and mprA-overexpressing strains at 5000×, 20,000×, and 40,000×. Insets show small portions of cell cross sections for each image. L-Glutamate and L-alanine fermentation by the WT/pXMJ19 (blue line) and WT/pXMJ19-*mprA* (cyan line) strains. OD_600_ and glucose consumption (**C**) and glutamate and alanine titers (**D**) were detected. The data are presented as mean ± SD of three independent biological replicates.

Next, fluorescence microscopy analysis of cells stained with Nile Red (lipid components) and Hoechst 33342 (DNA) revealed that the *mprA*-overexpressing strain showed elongated cell length and a swollen cell shape, whereas the cell morphology of the △*mprAB* mutant strain was similar to the wild-type strain (Figure 4C). This morphological heterogeneity was analyzed by scanning electron microscopy (SEM); 1.89 ± 0.31 μm for the wild-type strain, 1.87 ± 0.34 μm for the WT △*mprAB* mutant strain, and 4.45 ± 1.69 μm for the WT/pXMJ19-*mprA* strain. This indicates that cell elongation was caused by *mprA* overexpression (Figure 5A,B). In addition, wrinkles were observed on the cell surface of the *mprA*-overexpressing strain, suggesting potential cell membrane damage, leading to increased cytoplasmic membrane permeability and the release of cell plasma [43]. These findings were further confirmed by propidium iodide (PI) staining and flow cytometric single-cell analysis (Figure S3). Moreover, TEM revealed a more atypical cell shape in the *mprA-*overexpressing strain (Figure 5B). Furthermore, the fur-like structure of the cell wall surface suggests that the outer layer of the cell envelope may be composed of mycolic acid or arabinogalactan, which is partially absent in the WT/pXMJ19-*mprA* strain (Figure 5B).

Previous studies have indicated that disruption of cell envelope biosynthesis affects the excretion of amino acids in *Corynebacterium glutamicum* [44]. The WT/pXMJ19 and WT/pXMJ19-*mprA* strains were investigated for glutamate and alanine production in batch fermentation. As expected, the WT/pXMJ19-*mprA* strain exhibited slower growth and glucose consumption rates compared to the wild-type strain (Figure 5C). Within 72 h of fermentation, the WT/pXMJ19 strain exhibited near-complete glucose utilization (97.3 g/L), accompanied by the production of 666.3 mg/L glutamate and 522.4 mg/L alanine, whereas the WT/pXMJ19-*mprA* strain retained 7.3 g/L residual glucose and showed altered amino acid profiles (320.1 mg/L glutamate, 805.6 mg/L alanine) (Figure 5D). It is noteworthy that, compared to the WT/pXMJ19 strain, the glutamate titers in the WT/pXMJ19-*mprA* strain gradually decreased after 24 h, while the alanine titers continued to increase over 72 h. The observed metabolic flux redirection toward alanine biosynthesis in the WT/pXMJ19-*mprA* strain is potentially attributable to transaminase-mediated nitrogen transfer, wherein glutamate serves as the amino group donor [45].

### 3.5. The MprAB Two-Component System and Its Direct Target Gene htrA Are Induced by Vancomycin

**Figure 6:**
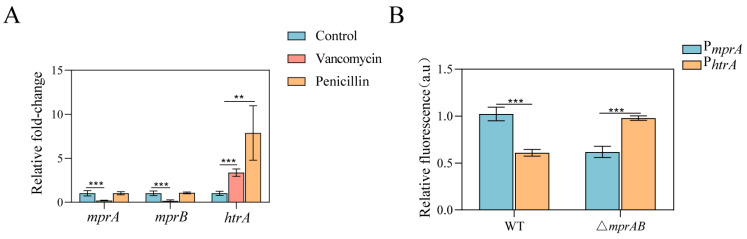
The MprAB TCS and HtrA are induced by the cell wall–active antibiotics vancomycin or penicillin. Transcript levels were assayed before (blue) and 1 h after the addition of 4 μg/mL vancomycin or 2 U/mL penicillin in the exponential phase. (**A**) The levels of *mprA*, *mprB*, and *htrA* in the WT strains were determined using qRT-PCR. Transcript levels before induction in the control strain were taken as 1.0. (**B**) The WT and △*mprAB* mutant strains carrying P*_mprA_*-EGFP and P*_htrA_*-EGFP were transformed with the reporter vector pEC-X1-P*_mprA_* and pEC-X1- P*_htrA_* for 8 h. The relative fluorescence was evaluated by the GFP/RFP ratio. The data are presented as mean ± SD of three independent biological replicates. Statistical significance was determined by Student’s *t* test. **, *p* < 0.01, ***, *p* < 0.001.

The MprAB system regulates several genes involved in cell wall biosynthesis, and overexpression of *mprA* causes cell wall defects in *C. glutamicum*. We tested whether cell wall–active antibiotics that impair cell envelope integrity induced MprAB expression. In the presence of vancomycin stimulation, the transcriptional levels of *mprA* and *mprB* were remarkably downregulated, as determined by qRT-PCR (Figure 6A). However, the transcription levels of *mprA* and *mprB* remained unaltered with the addition of penicillin. To investigate the activity of the Mpr regulon, a bicistronic plasmid containing the *egfp* gene under the control of the *mprA* promoter was constructed. To avoid the possible effect of plasmid copy number, the transcript level of red fluorescent protein (RFP) under the control of the constitutive promoter H_36_ on the same plasmid was determined by measuring the red fluorescence intensity [46]. The activities of the test promoters were easily and accurately evaluated using the GFP/RFP ratio. The promoter activity of the △*mprAB* mutant strain was decreased compared with that of the wild-type strain, which demonstrates positive autoregulation of expression of the MprAB system (Figure 6B).

Among the MprA target genes identified, *htrA* encodes a putative HtrA-like serine protease, which is involved in the response to protein secretion stress [47,48]. In *M. tuberculosis*, the HtrA homolog Pep*D* plays a key role in the cell envelope stress response [49]. To determine whether cell envelope stress induces the MprA-regulated expression of *htrA*, we measured *htrA* transcript levels in the wild-type and △*mprAB* mutant strains in the presence or absence of vancomycin and penicillin by qRT-PCR. As shown in Figure 6A, treatment of wild-type cells with vancomycin and penicillin resulted in significantly increased expression of *htrA* relative to untreated cells. Moreover, the expression of *htrA* was increased in the WT △*mprAB* mutant strain (Figure 6B). These results indicate that the expression of MprAB TCS and its direct target gene, *htrA*, respond to vancomycin.

### 3.6. HtrA Mediates Vancomycin and Penicillin Resistance, but MprAB Does Not

**Figure 7:**
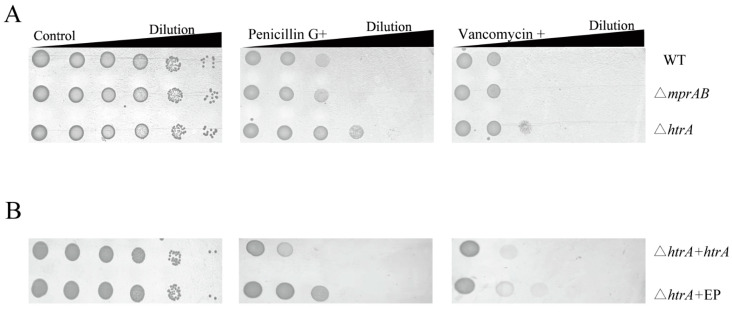
The wild-type strain and the indicated deletion mutants were grown, serially diluted, and spotted onto LBB plates containing either (**A**) 0.2 U/mL penicillin and 0.25 μg/mL vancomycin or (**B**) 0.2 U/mL penicillin, 0.25 μg/mL vancomycin, and 10 μg/mL chloramphenicol. EP: pXMJ19 empty plasmid.

*M. tuberculosis* MprAB and PepD have been reported to be required for resistance to cell envelope stress [14,49]. We therefore evaluated whether the MprAB TCS and HtrA are also essential for cell wall–active antibiotics resistance. The WT/pXMJ19-*mprA* strain was more sensitive to vancomycin and penicillin, and the WT/pXMJ19-*htrA* strain was more resistant to vancomycin, than the wild-type strain (Figure S4). However, there was no significant difference in the growth of wild-type and △*mprAB* mutant in penicillin and vancomycin resistance (Figure 7A). Thus, the MprAB TCS is responsive to, but does not mediate, vancomycin resistance in *C. glutamicum*. However, the △*htrA* mutant showed significantly better growth in the presence of penicillin and vancomycin compared to the wild-type strain (Figure 7A). Complementation by plasmid-encoded HtrA restored growth to wild-type levels (Figure 7B). Remarkably, the growth differences between the △*htrA* mutant and the △*htrA*/pXMJ19 mutant may be attributed to the presence of chloramphenicol. Thus, the MprAB TCS does not facilitate resistance to the cell envelope stressors vancomycin and penicillin, while its regulated gene *htrA* plays a critical role in mediating resistance to these stressors in *C. glutamicum*.

## 4. Discussion

The MprAB TCS is widely distributed in the genome of Actinobacteria, but its function and regulatory network have only been reported in mycobacteria. The cell envelope stress–responsive MprAB TCS, together with the alternative sigma factor SigE, forms a stress response network that facilitates the survival of tubercle bacilli inside host immune cells when they encounter nutrient and oxygen limitations and antibacterial mechanisms [16]. Characterization of the MprAB TCS in non-pathogenic *Corynebacterium* is critical for developing a complete knowledge of its conserved functions in Actinobacteria. In this study, we characterized the physiological functions and regulatory network of the MprAB TCS in *C. glutamicum*. Our results indicate that the MprAB TCS regulates a wide range of cellular pathways in response to impaired integrity of the cell envelope. RNA-seq analysis revealed only a slight perturbation of global transcript abundance in the △*mprA* mutant strain compared to the wild-type strain, suggesting that the MprAB TCS appears to be “off” under normal growth conditions. However, overexpression of *mprA* resulted in an intensely altered physiological status and transcript abundance in *C. glutamicum*. Several genes encoding secretory proteins (*cg0793*, *cg0107*, *cg2069*, *psp*2) and cell envelope biosynthesis proteins were significantly downregulated in the *mprA*-overexpressing strain (Table 1). One possible explanation is that these genes are involved in cell division, and a decrease in their expression may lead to morphological elongation. *cg0793*, encoding a cysteine-rich secretory protein with a CAP (cysteine-rich secretory protein) domain, is significantly regulated by MprA, as evidenced by a 3.45-fold increase upon *mprA* deletion and a significant decrease by 4.35-fold upon *mprA* overexpression. These protein families are most often involved in processes including extracellular matrix regulation and branching morphogenesis, potentially as either proteases or protease inhibitors. Cg0793 has been annotated as a hydrolase; however, its function is currently unclear. Considering the functionality of the MprAB TCS, we compared the growth of WT △*cg0793* mutant and wild-type strains on agar plates supplemented with antibiotics affecting cell wall activity. However, no phenotypic differences were detected under these test conditions, and the function of *cg0793* requires further investigation.

The purified MprA protein bound directly upstream of at least nine transcriptional units involved in multiple metabolic pathways, some with unknown functions. MEME analysis of the promoter regions of these regulatory sequences revealed an MprA-binding motif composed of a 10 bp flexible sequence (TKTTAAGAAM). In addition, the putative MprA-binding sites in the *mprA* promoter region, together with the P*_mprA_*-*egfp* reporter fusions, resulted in decreased relative activity in the *mprAB* deletion strain, suggesting positive autoregulation of the MprAB TCS. In mycobacteria, the MprAB TCS positively regulates *sigE* and *sigB* expression in response to cell envelope stress, and the absence of the regulator *mprA* results in downregulation of the expression of these genes [14]. In contrast, *sigE* and *sigB* expression in the △*mprA* mutant strain was comparable to that in the wild-type strain, whereas *mprA* overexpression directly repressed the transcript levels of *sigE* and *sigB* in *C. glutamicum*. Based on these findings, it is likely that an environmental signal is required for phosphorylation of the regulator MprA to control *sigE and sigB* expression in *C. glutamicum*. Thus, finding an appropriate extracellular stimulus to push the transition of MprAB TCS from the “off” to the “on” state may help to gain a more comprehensive understanding of the regulatory network between MprAB TCS and *sigE* and *sigB* in *C. glutamicum*.

Compared with *C. glutamicum* wild-type, an *mprA*-overexpressing mutant strain showed cell envelope defects and enhanced alanine titers. The enhanced alanine excretion could result from both impaired biosynthesis of cell envelope components, such as mycolic acids or peptidoglycan, and downregulation of alanine transporter Cg0524 expression. However, the loss of function of the MprAB TCS did not affect the growth of *C. glutamicum* under test conditions, including SDS, alkaline pH, and cell wall–active antibiotics. A different situation has been reported for *M. tuberculosis* and *Mycobacterium smegmatis,* which possess the MprAB TCS. In these cases, the *mprAB* TCS deletion strains were more sensitive to SDS than the wild-type strain. PepD not only is reported to be important in the stress response network mediated by MprAB in mycobacteria, but also its orthologous gene *htrA* can respond to periplasmic protein secretion stress in *C. glutamicum*, suggesting that HtrA family members maybe possess a conserved function in response to cell envelope stress in actinobacteria. Indeed, the promoter activity of *htrA* was MprAB-dependent, and *htrA* is responsive to vancomycin and penicillin. Compared to *C. glutamicum* wild-type, a △*htrA* mutant showed significantly increased growth on agar plates containing penicillin and vancomycin. These results clearly indicate that HtrA mediates penicillin and vancomycin resistance. In contrast, inactivation of *pepD* in *M. smegmatis* increases sensitivity to various cell wall stressors, but inactivation of *pepD* in *M. tuberculosis* is identical to wild-type with SDS-mediated stress [49].

Taken together, our results provide important novel insights into the transcriptional regulation and function of *C. glutamicum* MprAB TCS, indicating that it is clearly distinct from the *M. tuberculosis* MprAB TCS. (i) The MprAB TCS negatively affects the expression of genes that encode cell envelope functions and alternative sigma factors in *C. glutamicum*. (ii) Deletion of *mprAB* resulted a growth phenotype identical to *C. glutamicum* wild-type on agar plates containing cell envelope stressors. These functional differences may reflect *C. glutamicum*’s environmental adaptation strategy. The survival environments of non-pathogenic *C. glutamicum* fundamentally differ from those of pathogenic *M*. *tuberculosis*. *C. glutamicum* prioritizes the maintenance of cell envelope homeostasis to sustain rapid proliferation and high metabolic flux toward product synthesis. The MprAB TCS suppresses the expression of genes encoding cell envelope functions and alternative sigma factors, thereby preventing energy exhaustion caused by excessive cell envelope stress response. In contrast, *M*. *tuberculosis* requires continuous activation of cell envelope stress response mechanisms to survive harsh host environments. A future challenge is to elucidate how the activity of this system is controlled and how this TCS regulates the cell envelope process and function under diverse environmental stresses.

## Figures and Tables

**Table 1:** Significantly regulated genes, as determined by RNA-seq or qRT-PCR.

Gene Locus	Gene	Function	Binding Site ^a^	△*mprA* ^b^	*p-*Value ^c^	△*mprA/*pXMJ19*-mprA* ^d^	*p-*Value ^c^
cg0996	*mprA*	Response regulator of two-component system	TTTTAAGAGA	−3.65 ± 0.17	6.29 × 10^−101^	11.11 ± 0.75	4.28 × 10^−50^
cg0997	*mprB*	Histidine kinase of two-component system		−4.52 ± 0.17	3.91 × 10^−148^	−0.82 ± 0.35	2.05 × 10^−02^
Cell envelope biosynthesis							
cg0413	*cmt1*	Trehalose corynomycolyl transferase		−0.03 ± 0.29	9.17 × 10^−01^	−1.12 ± 0.08	3.22 × 10^−45^
cg0414	*wzz*	Saccharide synthesis		0.01 ± 0.13	9.51 × 10^−01^	−1.37 ± 0.08	2.64 × 10^−74^
cg0650		L,D-transpeptidases		0.01 ± 0.13	9.51 × 10^−01^	−1.62 ± 0.09	6.34 × 10^−77^
cg0905	*psp*2	Protein potentially involved in peptidoglycan biosynthesis		0.21 ± 0.41	6.10 × 10^−01^	−3.07 ± 0.14	4.18 × 10^−102^
cg0998	*htrA*	HtrA-like serine protease	TTTTCAGCAA	1.00 ± 0.13	1.75 × 10^−15^	−0.93 ±0.08	9.58 × 10^−29^
cg1108	*porA*	Mycoloyltransferase		0.08 ± 0.12	4.81 × 10^−01^	−1.45 ± 0.07	5.92 × 10^−94^
cg1109	*porB*	Mycoloyltransferase		1.48 ± 0.11	9.33 × 10^−43^	−0.74 ± 0.04	1.13 × 10^−71^
cg2069		Protein of LGFP repeat family		0.84 ± 0.19	8.46 × 10^−06^	−2.67 ± 0.15	3.69 × 10^−67^
cg2398	*plsC*	1-Acyl-sn-glycerol-3-phosphate acetyltransferase		0.00 ± 0.12	9.85 × 10^−01^	−1.16 ± 0.09	4.84 × 10^−40^
cg2478	*bla*	Penicillin-binding protein	TGTTAAGAAA	0.21 ± 0.14	1.20 × 10^−01^	−1.31 ± 0.10	5.11 × 10^−38^
cg3197	*csp*	Protein potentially involved in peptidoglycan biosynthesis	TGTTAAGAGC	0.00 ± 0.12	9.80 × 10^−01^	−2.00 ± 0.08	2.76 × 10^−144^
Regulatory proteins							
cg0317		ArsR family transcriptional regulator		−1.11 ± 0.37	2.99 × 10^−03^	1.44 ± 0.49	3.54 × 10^−03^
cg1119		Putative stress-responsive transcriptional regulator		0.31 ± 0.21	1.37 × 10^−01^	−1.38 ± 0.39	4.16 × 10^−04^
cg1271	*sigE*	Sigma factor	TGTGAAGTAC	−0.14 ± 0.15 (0.40 ± 0.10)	3.50 × 10^−01^	−0.10 ± 0.10 (−1.24 ± 0.05) ^e^	3.17 × 10^−01^
cg2102	*sigB*	Sigma factor	TGTTAAGAAC	−0.29 ± 0.20 (0.35 ± 0.25)	1.42 × 10^−01^	−0.11 ± 0.10 (−1.0 ± 0.07) ^e^	2.96 × 10^−01^
cg2115	sugR	Transcriptional regulators of sugar metabolism		−0.06 ± 0.13	6.52 × 10^−01^	−1.05 ± 0.11	1.74 × 10^−21^
cg2500	znr	Putative transcriptional regulator		−0.92 ± 0.36	9.44 × 10^−03^	0.64 ± 0.14	3.33 × 10^−06^
cg2648		Bacterial regulatory protein		3.83 ± 1.32	3.78 × 10^−03^	−1.02 ± 1.30	3.80 × 10^−02^
Secreted							
proteins							
cg0085	*porH1*	PhoH-like ATPase		0.12 ± 0.14	3.99 × 10^−01^	−1.36 ± 0.13	1.68 × 10^−26^
cg0625		Secreted protein	TTTTAAGGAC	−0.42 ± 0.20	3.27 × 10^−02^	1.10 ± 0.09	7.50 × 10^−33^
cg0726		Secreted lipoprotein		0.22 ± 0.14	1.25 × 10^−01^	−1.05 ± 0.14	1.72 × 10
cg0793		Secreted protein	AGCTAAGAAA ATTCTAAGAA	3.45 ± 0.19	4.35 × 10^−76^	−4.15 ± 0.29	2.30× 10^−45^
cg0918		Putative secreted protein		−0.03 ± 0.10	7.29 × 10^−01^	−1.31 ± 0.48	6.23 × 10^−03^
cg1247		Putative secreted protein		0.10 ± 0.58	8.67 × 10^−01^	−1.10 ± 0.09	4.42 × 10^−32^
cg1514		Putative secreted protein		−0.89 ± 0.25	3.17 × 10^−04^	1.02 ± 0.13	6.94 × 10^−15^
cg1936		Putative secreted protein		−0.09 ± 0.28	7.44 × 10^−01^	−1.16 ± 0.39	2.58 × 10^−03^
cg2061		Putative secreted protein		−0.25 ± 0.21	2.26 × 10^−01^	−2.93 ± 0.10	6.91 × 10^−177^
cg2518		Putative secreted protein		0.12 ± 0.17	4.69 × 10^−01^	−2.60 ± 0.12	7.07 × 10^−113^
cg2566		Putative secreted protein		−0.06 ± 0.21	7.80 × 10^−01^	−2.07 ± 0.12	3.88 × 10^−68^
cg3197		Putative secreted protein		0.00 ± 0.12	9.80 × 10^−01^	−2.00 ± 0.08	2.76 × 10^−144^
cg3343		Putative secreted protein		0.42 ± 0.25	9.70 × 10^−02^	−2.90 ± 0.10	1.96 × 10^−180^
cg3394		Putative secreted protein		0.35 ± 0.27	1.98 × 10^−01^	−1.70 ± 0.20	2.73 × 10^−17^
Other metabolism							
cg0010		Hypothetical protein		0.06 ± 0.30	8.54 × 10^−01^	−1.19 ± 0.19	2.70 × 10^−10^
cg0088		Citrate transporter		−40 ± 0.32	2.15 × 10^−01^	1.00 ± 0.40	1.19 × 10^−02^
cg0096		Hypothetical protein		−0.44 ± 0.47	3.56 × 10^−01^	1.16 ± 0.46	7.20 × 10^−124^
cg0107		Putative integral membrane transport protein		−0.25 ± 0.60	6.78 × 10^−01^	−5.07 ± 0.23	1.08 × 10^−02^
cg0108		Sirtuin-type KDAC homologues		−0.08 ± 0.15	5.98 × 10^−01^	−1.48 ± 0.27	4.42 × 10^−08^
cg0133	*abgT*	Secondary transporter of the AbgT family		−0.08 ± 0.14	5.94 × 10^−01^	−2.03 ± 0.08	1.43 × 10^−137^
cg0134	*abgB*	Peptidase		−0.05 ± 0.16	7.75 × 10^−01^	−2.01 ± 0.09	4.58 × 10^−122^
cg0135		Putative inner membrane protein		0.07 ± 0.20	7.28 × 10^−01^	1.13 ± 0.16	8.57 × 10^−13^
cg0182	*tagA2*	DNA-3-methyladenine glycosylase I		−0.28 ± 0.30	3.63 × 10^−01^	−0.65 ± 0.05	1.40 × 10^−108^
cg0253		Flavodoxin reductase		0.28 ± 0.0.24	2.45 × 10^−01^	−1.04 ± 0.17	1.63 × 10^−09^
cg0254		Alanine symporter		−0.98 ± 0.15	1.42 × 10^−10^	−1.06 ± 0.08	5.51 × 10^−37^
cg0291		3,4-dioxygenase beta subunit		0.15 ± 0.10	1.10 × 10^−01^	1.16 ± 0.10	1.07 × 10^−22^
cg0391		UDP-glucose 4-epimerase		−0.07 ± 0.12	5.75 × 10^−01^	−1.97 ± 0.08	4.70 × 10^−123^
cg0415	*ptpA2*	Low molecular weight protein-tyrosine phosphatase		0.05 ± 0.12	6.65 × 10^−01^	−1.11 ± 0.09	1.62 × 10^−35^
cg0437		Membrane protein		0.22 ± 0.14	1.03 × 10^−01^	−1.19 ± 0.12	5.66 × 10^−24^
cg0441	*lpd*	Dihydrolipoyl dehydrogenase		0.04 ± 0.14	7.70 × 10^−01^	−1.31 ± 0.09	6.20 × 10^−48^
cg0623		Cobalt transport system		−0.12 ± 0.19	5.28 × 10^−01^	1.10 ± 0.08	7.30 × 10^−43^
cg0646		IclR family proteins		0.12 ± 0.19	5.09 × 10^−01^	−1.93 ± 0.15	7.33 × 10^−38^
cg0692		Transposase		1.37 ± 0.58	1.77 × 10^−02^	0.51 ± 0.41	2.13 × 10^−01^
cg0703	*guaA*	GMP synthase		0.21 ± 0.41	6.10 × 10^−01^	−1.42 ± 0.63	2.49 × 10^−02^
cg0721	*crtB2*	Hytoene synthetase		0.2 ± 0.20	9.26 × 10^−01^	−1.11 ± 0.21	1.82 × 10^−07^
cg0723	*crtE*	Geranylgeranyl diphosphate synthase		0.03 ± 0.18	8.69 × 10^−01^	−1.06 ± 016	7.74 × 10^−11^
cg0727		Nucleoside-diphosphate-sugar epimerase		0.16 ± 0.12	1.81 × 10^−01^	−1.17 ± 0.11	1.76 × 10^−24^
cg0739		Putative integral membrane protein		0.06 ± 0.15	6.81 × 10^−01^	−1.42 ± 0.16	1.51 × 10^−18^
cg0740		Membrane protein		0.13 ± 0.18	4.85 × 10^−01^	−1.23 ± 0.13	1.53 × 10^−21^
cg0755	*metY*	O-Acetylhomoserine-lyase		0.03 ± 0.12	7.96 × 10^−01^	−1.05 ± 0.13	1.53 × 10^−21^
cg0770		Fe3+-siderophores transport system		−1.18 ± 0.12	1.32 × 10^−22^	−1.57 ± 0.14	6.58 × 10^−31^
cg0794	*ycic*	Cobalamin synthesis protein		3.27 ± 0.14	7.50 × 10^−123^	−3.42 ± 0.11	3.91 × 10^−195^
cg0830		Membrane protein		0.58 ± 0.27	3.23 × 10^−02^	−1.33 ± 0.45	3.05 × 10^−03^
cg0844		Type II restriction enzyme		−0.59 ± 0.17	4.15 × 10^−04^	−1.09 ± 0.09	9.28 × 10^−35^
cg0845		Superfamily II DNA/RNA helicase		−0.17 ± 0.22	4.26 × 10^−01^	−1.27 ± 0.13	2.73 × 10^−21^
cg0923		Membrane protein		0.15 ± 0.15	6.02 × 10^−01^	−1.14 ± 0.22	2.44 × 10^−07^
cg0924		Fe3+-siderophores transport system		−0.09 ± 0.09	7.50 × 10^−01^	−1.04 ± 0.16	4.79 × 10^−11^
cg1016	*betP*	Glycine betaine transporter		0.09 ± 0.21	6.72 × 10^−01^	−1.05 ± 0.11	1.62 × 10^−11^
cg1018	*recQ*	ATP-dependent DNA helicase		−0.28 ± 0.16	8.29 × 10^−02^	1.20 ± 0.08	2.81 × 10^−51^
cg1019		Metal-dependent hydrolase		0.06 ± 0.19	7.77 × 10^−01^	1.21 ± 0.36	8.24 × 10^−04^
cg1055	*menG*	2-demethylmenaquinone methyltransferase		0.08 ± 0.19	6.71 × 10^−01^	1.31 ± 0.09	1.54 × 10^−47^
cg1061	*urtA*	Urea transport system substrate-binding protein		1.09 ± 0.47	1.96 × 10^−02^	−0.52 ± 0.27	5.50 × 10^−02^
cg1077		Permease of the major facilitator superfamily		0.18 ± 0.15	2.30 × 10^−01^	−1.22 ± 0.19	5.56 × 10^−11^
cg1088		ABC-type multidrug/protein/lipid transport system		0.22 ± 0.17	1.83 × 10^−01^	−1.35 ± 0.08	1.67 × 10^−60^
cg1178		Transposase		−2.43 ± 0.27	1.69 × 10^−19^	−0.61 ± 0.21	4.00 × 10^−03^
cg1182		Putative membrane protein		−1.97 ± 0.19	3.55 × 10^−24^	−0.20 ± 0.16	2.20 × 10^−01^
cg1183		Predicted dinucleotide-utilizing enzyme		−2.15 ± 0.21	1.78 × 10^−25^	−0.13 ± 0.15	3.89 × 10^−01^
cg1184		Transposase		−1.80 ± 0.20	8.97 × 10^−20^	−0.13 ± 0.19	3.89 × 10^−01^
cg1244		Arsenate reductase or related protein		0.17 ± 0.39	6.55 × 10^−01^	−1.37 ± 0.50	6.53 × 10^−03^
cg1305		Amino acid permease		−0.09 ± 0.19	6.20 × 10^−01^	−1.15 ± 0.21	6.84 × 10^−08^
cg1427		Extracellular deoxyribonuclease		0.43 ± 0.25	8.64 × 10^−02^	1.45 ± 0.12	3.40 × 10^−35^
cg1438		ATPase component		1.83 ± 0.93	4.98 × 10^−02^	−0.27 ± 0.24	1.54 × 10^−01^
cg1493		D-alanine--d-alanine ligase A		0.00 ± 0.13	8.67 × 10^−01^	−1.07 ± 0.10	2.12 × 10^−29^
cg1551	*uspA1*	Universal stress protein		0.12 ± 0.11	2.71 × 10^−01^	−1.13 ± 0.08	1.18 × 10^−50^
cg1642		Siderophore-interacting protein		−1.02 ± 0.49	3.80 × 10^−02^	1.33 ± 0.40	8.43 × 10^−04^
cg2043		Hypothetical protein		−0.02 ± 0.13	8.98 × 10^−01^	−1.01 ± 0.08	5.60 × 10^−37^
cg2181		ABC-type peptide transport system		−0.59 ± 0.96	5.40 × 10^−01^	−1.62 ± 0.26	5.46 × 10^−10^
cg2343		Decarboxylase		0.03 ± 0.27	9.24 × 10^−01^	−1.05 ± 0.08	5.25 × 10^−40^
cg2358		Hypothetical protein		−0.19 ± 0.40	6.25 × 10^−01^	−1.33 ± 0.31	1.47 × 10^−05^
cg2359		Isoleucine-tRNA ligase-like protein		−0.16 ± 0.13	1.96 × 10^−01^	−1.21 ± 0.09	1.92 × 10^−39^
cg2386		Hypothetical protein		−0.27 ± 0.28	3.36 × 10^−01^	−1.01 ± 0.14	1.05 × 10^−12^
cg2397		Putative membrane protein		0.04 ± 0.11	7.07 × 10^−01^	−1.49 ± 0.10	9.83 × 10^−02^
cg2565		Hypothetical protein		−0.14 ± 0.13	3.03 × 10^−01^	−2.45 ± 0.11	1.09 × 10^−103^
cg2651		Hypothetical protein		1.37 ± 0.26	1.96 × 10^−07^	−3.71 ± 0.13	1.29 × 10^−187^
cg2662	*pepN*	Aminopeptidase		−0.19 ± 0.14	1.65 × 10^−01^	−1.44 ± 0.09	8.29 × 10^−53^
cg2807	*tnp11a*	Transposase		0.31 ± 0.21	1.31 × 10^−01^	−1.04 ± 0.15	2.78 × 10^−12^
cg2844	*pstA*	ABC-type phosphate transport system		−0.11 ± 0.26	6.65 × 10^−01^	−1.12 ± 0.16	9.03 × 10^−12^
cg2845	*pstC*	ABC-type phosphate transport system		−0.28 ± 0.24	2.39 × 10^−01^	−1.21 ± 0.12	2.31 × 10^−19^
cg2846	*pstS*	ABC-type phosphate transport system		−0.26 ± 0.24	2.80 × 10^−01^	−1.36 ± 0.12	1.97 × 10^−29^
cg2870	*dctA*	Na+/H+-dicarboxylate symporter		0.50 ± 0.20	1.16 × 10^−02^	−1.47 ± 0.13	1.46 × 10^−28^
cg2895		Permease of the major facilitator superfamily		−0.00 ± 0.13	2.80 × 10^−01^	−2.43 ± 0.12	2.59 × 10^−92^
cg2896		Endoglucanase		−0.05 ± 0.14	7.52 × 10^−01^	−1.36 ± 0.12	1.97 × 10^−29^
cg3086		L,L-Cystathionine gamma-Lyase		−0.21 ± 0.18	2.36 × 10^−01^	−1.02 ± 0.20	1.93 × 10^−07^
cg3105		Hypothetical protein		−0.30 ± 0.17	7.70 × 10−02	1.04 ± 0.13	1.97 × 10^−29^
cg3106		Hypothetical protein		−0.13 ± 0.14	3.66 × 10^−01^	−1.14 ± 0.09	6.64 × 10^−37^
cg3270		Hypothetical protein		0.34 ± 0.30	2.53 × 10^−01^	−2.63 ± 0.40	7.68 × 10^−05^
cg3292		Copper chaperone		−0.07 ± 0.16	6.73 × 10^−01^	−1.19 ± 0.18	3.97 × 10^−11^
cg3395	*proP*	Proline/betaine transporter		0.34 ± 0.19	7.13 × 10^−02^	−2.37 ± 0.10	1.25 × 10^−122^
cg3403		Permease of the major facilitator superfamily		0.28 ± 0.30	3.60 × 10^−01^	−1.32 ± 0.44	2.75 × 10^−03^
cg3404		ABC-type transport system		−0.19 ± 0.30	5.26 × 10^−01^	−1.15 ± 0.21	2.89 × 10^−08^

^a^ The motif sequence of the MprA protein bound to these genes in the EMSA experiment. ^b^ The relative ratios of transcript level of genes of the *C. glutamicum* △*mprA* mutant strain vs. the *C. glutamicum* wild-type strain, as determined by RNA-seq, are shown as base 2 logarithm values. The data are presented as the mean ± SD of three independent biological replicates. ^c^ *p*-values were determined by Student’s t-test. ^d^ The relative ratios of transcript levels of genes in the *mprA-*overexpressing *C. glutamicum* △*mprA* mutant strain vs. the *C. glutamicum* △*mprA* mutant strain, as determined by RNA-seq and shown as base 2 logarithm values. The data are presented as the mean ± SD of three independent biological replicates. ^e^ The RNA-seq results were inconsistent with the qRT-PCR results, and the values in parentheses indicate the transcript levels measured by qRT-PCR. The data are presented as the mean ± SD of three independent biological replicates.

## Data Availability

The data used in this study can be accessed by request from the corresponding authors.

## Supplementary Materials

The following supporting information can be downloaded at: https://www.mdpi.com/article/10.3390/microorganisms13051120/s1, Figure S1: Verification of RNA-seq results using qRT-PCR; Figure S2: EMSA analysis of MprA_D-E_ and MprA_D-N_ binding to the promoter of *mprA*, *sigE*, *pepD*, *cg0793*; Figure S3: Microscopic pictures and flow cytometric single-cell analysis of *C. glutamicum* wild type (ATCC 13032) and the overproduction of *mprA* in the wide type; Figure S4: Antibiotics sensitivity of *C. glutamicum* wide-type and overexpressing strains. Table S1: Bacterial strains and plasmids used in this study; Table S2: Primers used in this study.
