# Correction: Zou et al. Two-Component MprAB System Regulates the Expression of Genes Involved in Cell Envelope Biosynthesis in *Corynebacterium glutamicum*. *Microorganisms* 2025, *13*, 1120

**DOI:** 10.3390/microorganisms14071576

**Published:** 2026-07-20

**Authors:** Yu Zou, Danni Huang, Xiuxia Liu, Yankun Yang, Chunli Liu, Ye Li, Zhonghu Bai

**Affiliations:** 1 School of Biotechnology and Key Laboratory of Industrial Biotechnology of Ministry of Education, Jiangnan University, Wuxi 214122, China; jndxzy1997@163.com (Y.Z.); 6200203003@stu.jiangnan.edu.cn (D.H.); yangyankun@jiangnan.edu.cn (Y.Y.); liuchunli@jiangnan.edu.cn (C.L.); liye@jiangnan.edu.cn (Y.L.); 2 National Engineering Research Center for Cereal Fermentation and Food Biomanufacturing, Jiangnan University, Wuxi 214122, China; 3 Jiangsu Provincial Engineering Research Center for Bioactive Product Processing, Jiangnan University, Wuxi 214122, China; 4 Zhengzhou University of Technology, Zhengzhou 450044, China

In the original publication [1], *pepD* was incorrectly described in the legend of Figure S2. The correct legend appears below.

The legend for Figure S2 should be described as follows: EMSA analysis of MprA_D-E_ and MprA_D-N_ binding to the promoter of *mprA*, *sigE*, *htrA*, *cg0793*.

In the original publication, there was a mistake in Figures 2B, S2C and 5D as published. The gene names for *cg3197* (*bla*), *cg2478* (*csp*), and *cg0794* were incorrectly described in Figure 2B. *pepD* was incorrectly described in Figure S2C. The colors do not accurately correspond to their respective data groups in Figure 5D. The corrected Figure 2B, Figure S2C and Figure 5D appears below.

**Figure 2:**
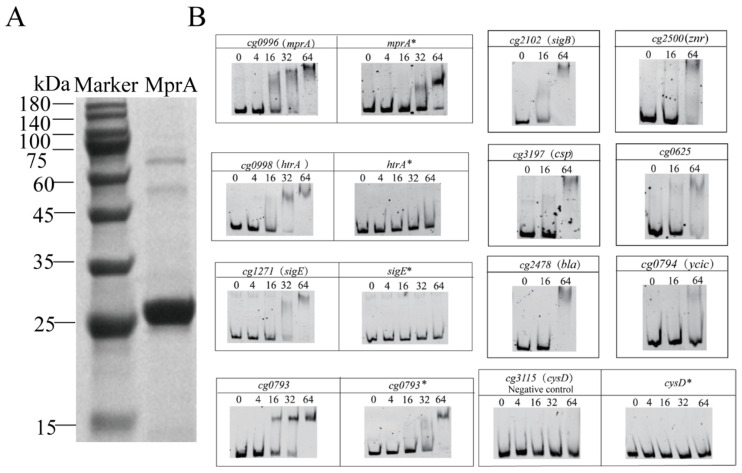
Electrophoretic mobility shift assays (EMSAs) were performed to identify binding of MprA to promoter regions. (**A**) SDS-PAGE of the MprA protein. (**B**) DNA fragments about 300 bp upstream of the putative MprA target genes were incubated with or without a 4, 16, 32, or 64 molar excesses of phosphorylated purified MprA protein, as indicated below the respective lanes. The promoter region of the untargeted gene *cysD* was used as a negative control. The asterisk (*) indicates samples without prior phosphorylation of MprA by acetylphosphate. No DNA binding was observed without previous phosphorylation of MprA with acetylphosphate (htrA*, sigE*, and cysD*).

**Figure S2:**
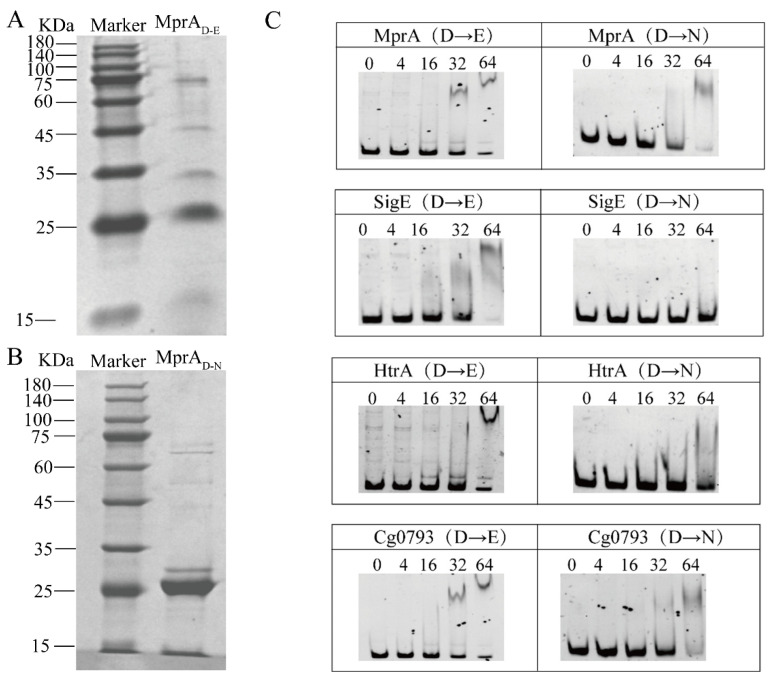
**EMSA analysis of MprA_D-E_ and MprA_D-N_ binding to the promoter of *mprA*, *sigE*, *htrA*, *cg0793*.** (**A**) Phosphorylation-mimic MprA_D-E_ containing a C-terminal His-tag was overproduced in *Escherichia coli* BL21 (DE3) and purified. (**B**) Phosphorylation-defective MprA_D-N_ containing a C-terminal His-tag was overproduced in *Escherichia coli* BL21 (DE3) and purified. (**C**) EMSA experiments were incubated for 30 min at 20 °C without or with a 4, 16, 32 and 64 molar excess of purified MprA_D-E_ and MprA_D-N_ protein as indicated below the respective lanes.

**Figure 5:**
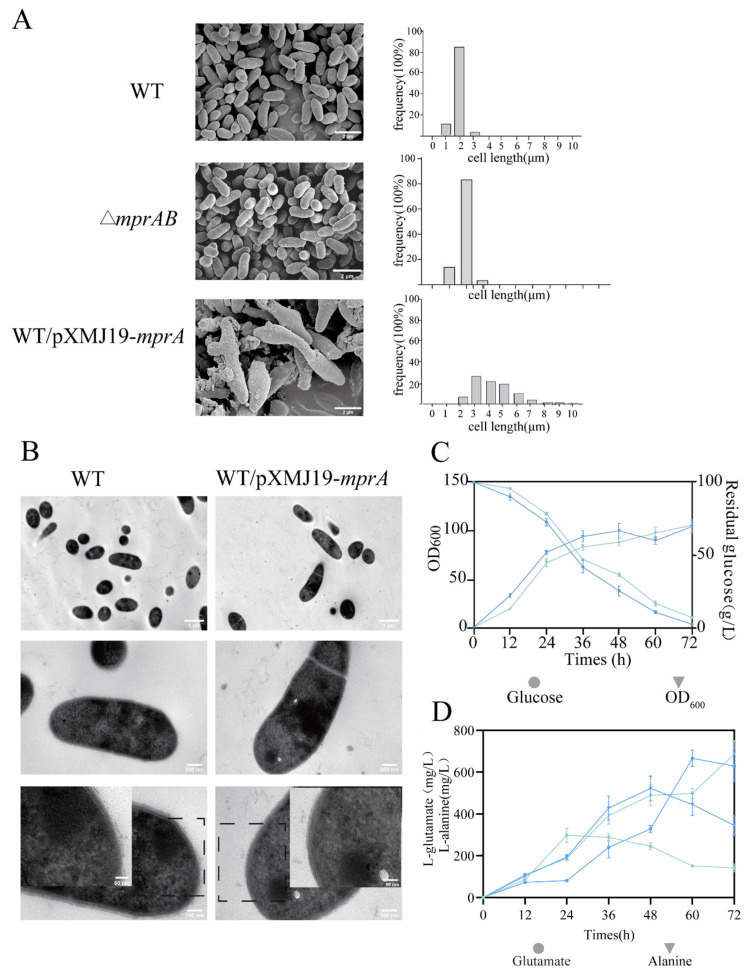
Physiological characteristics of *C. glutamicum* cells. For microscopic analysis, cells were cultured in CGXII minimal medium containing 4% glucose for 24 h, and *mprA* expression was induced by adding 1 mM IPTG. (**A**) FSEM micrographs of WT, Δ*mprAB* mutant, and *mprA*-overexpressing strains. FSEM pictures were captured at 10,000× magnification. Size distribution of WT, Δ*mprAB* mutant, and *mprA*-overexpressing strains. The lengths of at least 100 individual cells were measured. Cells were fixed using 3% glutaraldehyde. (**B**) TEM images of wild-type and mprA-overexpressing strains at 5000×, 20,000×, and 40,000×. Insets show small portions of cell cross sections for each image. L-Glutamate and L-alanine fermentation by the WT/pXMJ19 (blue line) and WT/pXMJ19-*mprA* (cyan line) strains. OD_600_ and glucose consumption (**C**) and glutamate and alanine titers (**D**) were detected. The data are presented as mean ± SD of three independent biological replicates.

There was an error in the original publication. The results were incorrectly described.

Following publication, a duplication was identified in Table 1, where “cg3197” was listed twice. The duplicate entry associated with the “regulatory proteins” category has been removed.

A correction has been made to Overexpression of *mprA* caused cell envelope defects and increased alanine titers in *C. glutamicum*, where the WT/pXMJ19-*mprA* strain retained 7.3 g/L residual glucose and showed altered amino acid profiles (297.8 mg/L glutamate and 686.9 mg/L alanine).

The authors state that the scientific conclusions are unaffected. This correction was approved by the Academic Editor. The original publication has also been updated.

## References

[1] Zou Y., Huang D., Liu X., Yang Y., Liu C., Li Y., Bai Z. (2025). Two-Component MprAB System Regulates the Expression of Genes Involved in Cell Envelope Biosynthesis in *Corynebacterium glutamicum*. Microorganisms.

