# Supplementary material for: Two-Component MprAB System Regulates the Expression of Genes Involved in Cell Envelope Biosynthesis in Corynebacterium glutamicum
Source: Microorganisms. 2025 May 13;13(5):1120. doi: 10.3390/microorganisms13051120 (PMC12113857; doi:10.3390/microorganisms13051120)
Supplement: Supplementary file 1 [file microorganisms-13-01120-s001.zip › microorganisms-3559639-supplementary.pdf]

## Table S1. Bacterial strains and plasmids used in this study.

| Strains or Plasmids | Description | Reference |
|---|---|---|
| *E. coli* | | |
| JM109 | endA1 recA1 gyrA96 thi-1 hsdR17 (rk-,mk+) relA1 supE44 D (lac-proAB) [F′ traD36 proAB laqI qZΔM15] (DE3) | Weidibio |
| BL21 (DE3) | F- ompT hsdS(rB-mB-) gald cm (DE3) | Weidibio |
| BL21 (DE3)/pET-28-*mprA*-CHis6 | BL21 (DE3) derivativec pET-28a-*mprA*-CHis6 | This study |
| BL21 (DE3)/pET-28-$mprA_{D\text{-}E}$-CHis6 | BL21 (DE3) derivative harboring pET-28-$mprA_{D\text{-}E}$-CHis6 | This study |
| BL21 (DE3)/pET-28-$mprA_{D\text{-}N}$-CHis6 | BL21 (DE3) derivative harboring pET-28-$mprA_{D\text{-}N}$-CHis6 | This study |
| *C. glutamicum* | | |
| ATCC 13032 | Wild-type strain, Biotin-deficient gentype | Lab stock |
| WT △*mprA* | ATCC 13032 has deletions in *mprA,* retaining the 45 bp N-terminal nucleotide sequence of *mprA.* | |
| WT △*mprA*/pXMJ19 | △*mprA* mutant harboring empty plasmid | This study |
| WT △*mprA*/pXMJ19-*mprA* | △*mprA* mutant harboring pXMJ19-*mprA* | This study |
| WT △*mprAB* | WT △*mprA* has deletions in *mprB*, retaining the 63 bp C-terminal nucleotide sequence of *mprB*. | This study |
| WT △*htrA* | ATCC 13032 has deletions in *htrA,* retaining the 21 bp N-terminal nucleotide sequence of *htrA* and the 30 bp C-terminal nucleotide sequence of *htrA*. | |
| WT/pXMJ19-*mprA* | ATCC 13032 harboring pXMJ19-*mprA* | This study |
| WT/pECX1-$P_{mprA}$ | ATCC 13032 harboring pECX1-$P_{mprA}$ | This study |
| WT△*mprAB*/pECX1-$P_{mprA}$ | △*mprAB* mutant harboring pECX1-$P_{mprA}$ | This study |
| Plasmids | | |
| pET-28a | $oriV_{E.coli}$, $Kan^R$, $P_{T7}$ LacI. | Novagen |
| pET-28-*mprA*-CHis6 | pXMJ19 derivative harboring *mprA*-CHis6 | This study |
| pET-28-$mprA_{D\text{-}E}$-CHis6 | pXMJ19 derivative harboring $mprA_{D\text{-}E}$-CHis6 | This study |
| pET-28-$mprA_{D\text{-}N}$-CHis6 | pXMJ19 derivative harboring $mprA_{D\text{-}N}$-CHis6 | This study |
| pK18mobsacB | *C. glutamicum*/*E. coli* shuttle vector ($oriV_{E.\ coli}$, *sacB*, *lacZα*, $Kan^R$) | 1 |
| pXMJ19 | *C. glutamicum*/*E. coli* shuttle vector ($Cm^R$, $P_{tac}$, $lacI^q$ , pBL1 $oriV_{C.g}$, $oriV_{E.coli}$) | 2 |
| pEC-X99KE | *C. glutamicum*/*E. coli* shuttle expression vector ($P_{trc}$, $lacI^q$, $Kan^R$) | 3 |
| pEC-X1-$P_{mprA}$ | pEC-X99KE derivative harboring gene cassette $P_{mprA}$-*egfp*-terminator-$H_{36}$-*mcherry* | This study |
| pEC-X1-$P_{htrA}$ | pEC-X99KE derivative harboring gene cassette $P_{htrA}$-*egfp*-terminator-$H_{36}$-*mcherry* | This study |
| pXMJ19-*mprA* | pXMJ19 derivative harboring *mprA* under the $P_{tac}$ | This study |
| pXMJ19-*htrA* | pXMJ19 derivative harboring *htrA* under the $P_{tac}$ | This study |
| pK18-komprA | *pK18mobsacB* harboring a 2.0-kb homologous fragment for *mprA* deletion | This study |
| pK18-komprB | *pK18mobsacB* harboring a 2.0-kb homologous fragment for *mprB* deletion | This study |
| pK18-kohtrA | *pK18mobsacB* harboring a 2.0-kb homologous fragment for *htrA* deletion | This study |

$Cm^R$: chloramphenicol-resistant; $Kan^R$: kanamycin-resistant.

**Table S2 Primers used in this study.**

| Primers | Sequence | Description |
|---|---|---|
| OPmprA-F | **TAAGAAGGAGATATACCATG**AAAATTTTAGTTGTTGATGACGAGCA | Purication of MprA protien |
| OPmprA-R | **CAGTGGTGGTGGTGGTGGTG**CGGAGCGGTCTCTCGCAG | |
| OpmprA$_{D-E}$-F | CATGACTTCGAGGATCACCAAAGC | Purication of MprA$_{D-E}$ protein |
| OpmprA$_{D-E}$-R | CTCGAAGTCATGATGCCTGGTATGG | |
| OpmprA$_{D-N}$-F | CATGACATTGAGGATCACCAAAG | Purication of MprA$_{D-N}$ protien |
| OpmprA$_{D-N}$-R | CTCAATGTCATGATGCCTGGTATGG | |
| pET-28-F | **CATGGTATATCTCCTTCTTAAA**GTTAAACA | |
| pET-28-R | **CACCACCACCACCACCACTG** | |
| ORmprA-F | **TTAAAGGAGGACAACTAATG**AAAATTTTAGTTGTTGATGACGAGCA | Overproudction of *mprA* |
| ORmprA-R | **CAGTTCCCTACTCTCGCATG**TTACGGAGCGGTCTCTCGC | |
| ORhtrA-F | **AGCTTAAAGGAGGACAACTAATG**ACAAATCAATTCCCCACAAA | Overproudction of *htrA* |
| ORhtrA-R | **GGCAGTTCCCTACTCTCGCATG**CTACTCACTCGTCAGAGTAACCTCTACC | |
| pXMJ19-F | **CATGCGAGAGTAGGGAACTGC**C | |
| pXMJ19-R | **CATTAGTTGTCCTCCTTTAAGCTTAA**TTAATTCTGTTTCCTG | |
| CpXMJ19-F | GGATGAGAGAAGATTTTCAGCCTG | |
| pXMJ19-R | **CATTAGTTGTCCTCCTTTAAGCTTAA**TTAATTCTGTTTCCTG | |
| ChtrA-F | **AGCTTAAAGGAGGACAACTA**ATGACAAATCAATTCCCCACAAA | Complementation of *htrA* |
| ChtrA-R | **CTGAAAATCTTCTCTCATCC**TTACTCACTCGTCAGAGTAACCTCTACC | |
| P$_{mprA}$-F | **GCGCGAGGCAGCAGATCAAT**TCTGGTTAAGGCAGCACAGCT | Determination of P$_{mprA}$ activity |
| P$_{mprA}$-R | **TCCTCGCCCTTGCTCACCAT**TAAGTAGACCTCCTTTTAAGGGAACGTCGCAAGGAG | |
| P$_{htrA}$-F | **GCGCGAGGCAGCAGATCAAT**ATAATGCCATTAAATGGTCGCC | Determination of P$_{htrA}$ activity |
| P$_{htrA}$-R | **ACTCCTTGCGACGTTCCCTTAAAAGGAGGTCTACTTA** GGAGTTGGTTTCTGATGGAGTTTC | |
| pECX1-F | **ATGGTGAGCAAGGGCGAGGA**GCT | |
| pECX1-R | **ATTGATCTGCTGCCTCGCG** | |
| KomprA-F1 | **ACGACGGCCAGTGCCAAGCT**TGCTCTGATGCGGTATGGATGTG | Deletion of *mprAB* |
| KomprA-R1 | **CTTAATGTCACGGAGTCACG**TACAGCTTGCTCG | |

| | | |
|---|---|---|
| KomprA-F2 | **CGTGACTCCGTGACATTAAG**GCGAATCGGC | |
| KomprA-R2 | **TATGACCATGATTACGAATT**CGCGCAATTTCCAACAC TTGG | |
| komprB-R1 | **GTGATTCTGGAGTCACGTAC**AGCTTGCTCG | |
| KomprB-F2 | **GTACGTGACTCCAGAATCAC**TATTGATTTGCCAGGG | |
| KomprB-R2 | **TATGACCATGATTACGAATT**CTCGGTCTGGATAGCAT CGATG | |
| KohtrA-F1 | **ACGACGGCCAGTGCCAAGCTT**TTGATCATTGGCGGC AGTG | Deletion of *htrA* in the wild-type background |
| KohtrA-R1 | **GTCAGAGTAACCTTTTCGTC**TGTGGGGAATTGATTT GTCATG | |
| KohtrA-F2 | **GACGAAAAGGTTACTCTGAC**GAGTGAG | |
| KohtrA-R2 | **CGAATTGCAATTACCGAATT**CTCCATCGAAGCAGAA CTCC | |
| cx-mprA-F | AAACAATGGCAGTTCCAAAG | |
| cx-mprA-R1 | GTTCGAAGGAGTTGGTTTCTG | |
| cx-mprAB-F | TTGACGTCACCAGCGAGTCT | |
| cx-mprAB-R | CATCTGTGCTTAGAGGCTGA | |
| cx-htrA-F | ACGGTCAGACCGTTCGCATC | |
| cx-htrA-R | GAACTCGTGTGTCTACAACATC | |
| QmprA-F | TCGCAGAAGACGGCATCCAA | Validation of qRT-PCR |
| QmprA-R | CAGAAACATTATCGCGGGCA | |
| QmprB-F | CTTTGCTGAGCGCCACTTTG | |
| QmprB-R | GGTTGCATAGAATCCCGCTT | |
| QsigE-F | AACATGCTGAAGAACTCACG | |
| QsigE-R | GGAAGACACGCATGAATGTT | |
| QsigB-F | GAACGTGAGGTAGATCCCGG | |
| QsigB-R | CTCTGCATAAAGGCCAACCT | |
| QhtrA-F | CCATCAGAAACCAACTCCTT | |
| QhtrA-R | ACATCTGTGCTTAGAGGCTG | |
| QclgR-F | CGCGTTCCAATTCTGAAAGA | |
| QclgR-R | AACCCTTCTAGACAAGCCG | |
| Qcg2500-F | CCTGCAGAAGAGTCCGAATC | |
| Qcg2500-R | GCTCACTAGTGGTTGCGAAC | |
| Qcg0793-F | GCTGTTTCCACCGCCATGA | |
| Qcg0793-R | GATGCTGGTTAACGAGCGCA | |
| MprA-F | CGACAATGTCGCCCTTCAG | EMSA experiments |
| MprA-R | CGATACCCTTTTCGTCTTCC | |
| SigE-F | TAATCGCGATGGCACCAG | |
| SigE-R | TTTGCCCCACTGAACTTG | |
| HtrA-F | AGGGCCTGGAATTGCTGAAA | |
| HtrA-R | GATTTGATTATGAACCCTGTGGC | |
| Cg0794-F | GTGAAAGCCCTCCTTTTGG | |
| Cg0794-R | GGGGTGCTCCTAAAAAGCGA | |

| | |
|---|---|
| Cg0793-F | AGAGGATCCGATCGAGAAG |
| Cg0793-R | TTGTCTCCTTGTGTCATTGC |
| Cg2500-F | ACCAGTCCACGTCGTTTAC |
| Cg2500-R | GGGGAAGTCCTTCCGTCC |
| CysD-F | TGTTGCGACTCACCTCTTTT |
| CysD-R | GCGTTGTTACCCACTTCAAG |
| GyrA-F | CATACTCGTGCAGCATCGTC |
| GyrA -R | TGATCACCTTTCTTCGTGTCAAC |
| Csp-F | TTTCGATTCCGTCATGCATTC |
| Csp-R | CGGTTTTTTCCGAGTCTCG |
| Bla-F | GGAGACAGACCTTAGCAACTAA |
| Bla-R | AACGACCCAGCAGAATTCGAC |
| SigB-F | ATCGTGGTCTGATGCAGCA |
| SigB-R | AACTGGCCTCCTAAATTCG |
| Cg0625-F | GAAAACATGTCCTTAAAATGAATTGG |
| Cg0625-R | CTTACATTTTCCCTGCCACC |
| -287cg0793-F | AGAGGATCCGATCGAGAAG |
| -287cg0793-R | CGCTGTGTCCGGCTGATT |
| -211cg0793-F | TTGATGAATCAGCCGGACAC |
| -211cg0793-R | GATCAAACAAGTTCAGCAACT |
| -134cg0793-F | GGAGTTGCTGAACTTGTTTG |
| -134cg0793-R | TGTTTTCTTAGCTCATTGCATTCC |
| -49cg0793-F | TGGGGAATGCAATGAGCTA |
| -49cg0793-R | TTGTCTCCTTGTGTCATTGC |
| -64cg0793-F | ATGAGCTAAGAAAACACTTTAAATATTCTA |
| -64cg0793-R | TAGAATATTTAAAGTGTTTTCTTAGCTCAT |
| -55cg0793-F | GAAAACACTTTAAATATTCTAA |
| -55cg0793-R | TTAGAATATTTAAAGTGTTTTC |
| -43cg0793-F | AATATTCTAAGAAACAGTTCAAGTTTTGCT |
| -43cg0793-R | AGCAAAACTTGAACTGTTTCTTAGAATATT |
| -5cg0793-F | GATATGACAAGGCAATGACACAAGGAGACA |
| -5cg0793-R | TGTCTCCTTGTGTCATTGCCTTGTCATATC |

Bold: the homology arm.

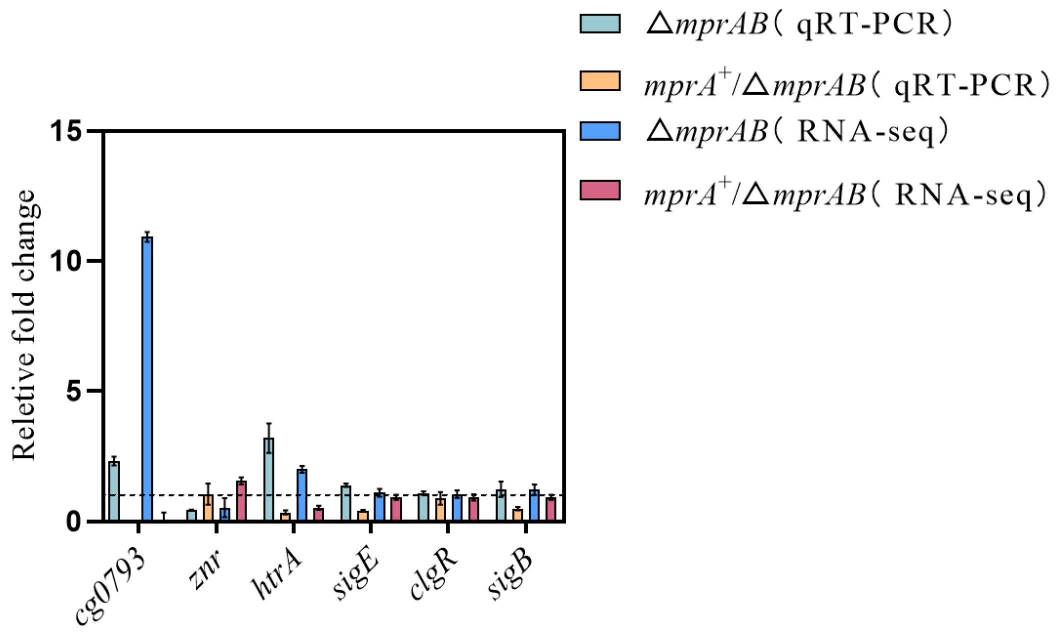


**Figure S1 Verification of RNA-seq results using qRT-PCR.** RNA-seq datas were verified with qRT-PCR. The transcript level of each gene in the wild-type and △*mprA* carrying the vectors were set at 1.0, respectively. Error bars represent the SD from three biological independent experiments.

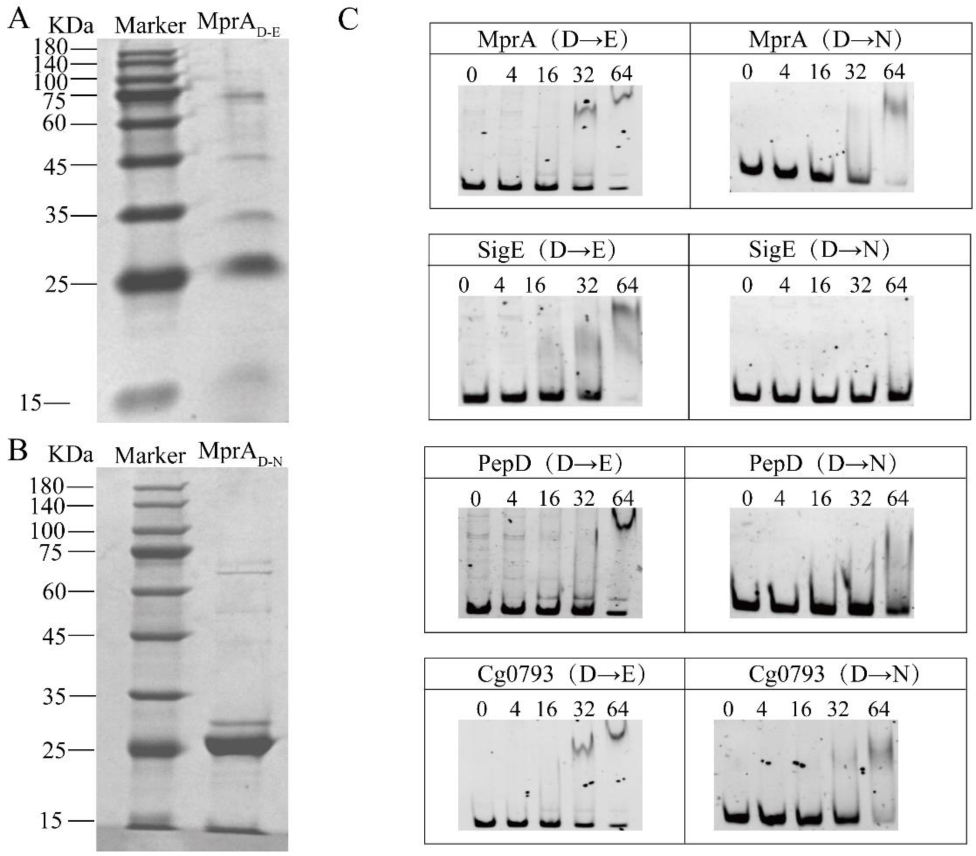


**Figure S2 EMSA analysis of $\mathbf{MprA_{D\text{-}E}}$ and $\mathbf{MprA_{D\text{-}N}}$ binding to the promoter of *mprA*, *sigE*, *pepD*, cg0793. (A)** Phosphorylation-mimic $MprA_{D\text{-}E}$ containing a C-terminal His-tag was overproduced in *Escherichia coli* BL21 (DE3) and purified. (B) Phosphorylation-defective $MprA_{D\text{-}N}$ containing a C-terminal His-tag was overproduced in *Escherichia coli* BL21 (DE3) and purified. (c) EMSA experiments were incubated for 30 min at 20 °C without or with a 4, 16, 32 and 64 molar excess of purified $MprA_{D\text{-}E}$ and $MprA_{D\text{-}N}$ protein as indicated below the respective lanes.

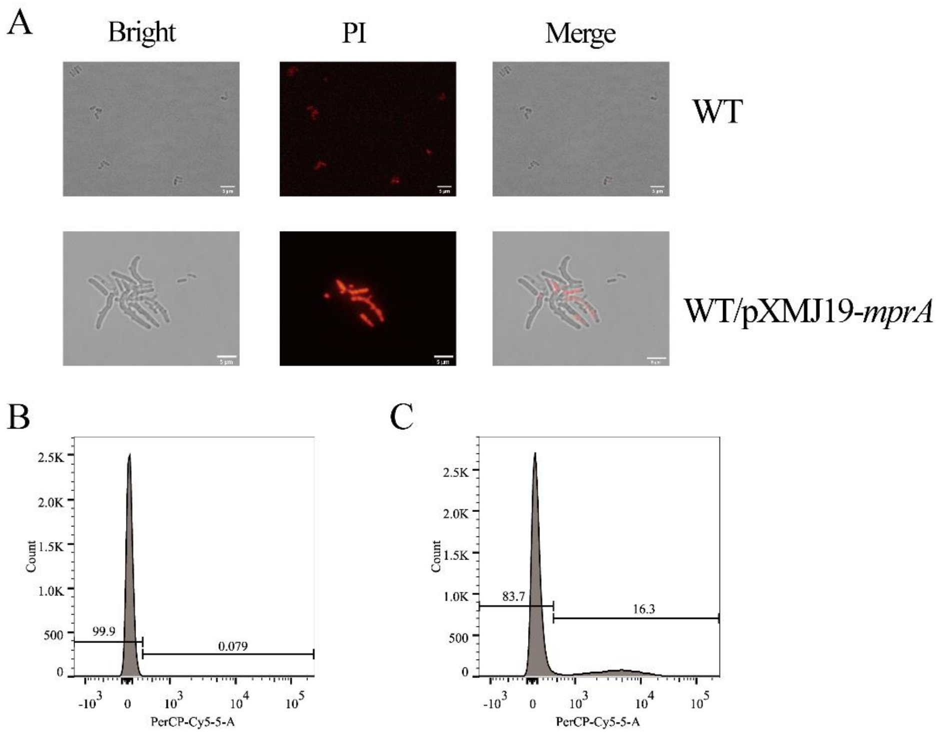


**Figure S3 Microscopic pictures and flow cytometric single-cell analysis of *C. glutamicum* wild type (ATCC 13032) and the overproduction of *mprA* in the wide type. (A)** The cultures were grown in the CGXII medium containing 4% glucose at 24 h with PI staining of dead cell. (B) Propidium iodide staining using flow cytometric single-cell analysis of wild type/pXMJ19. (C) Propidium iodide staining using flow cytometric single-cell analysis of *mprA* overexpressing strain.

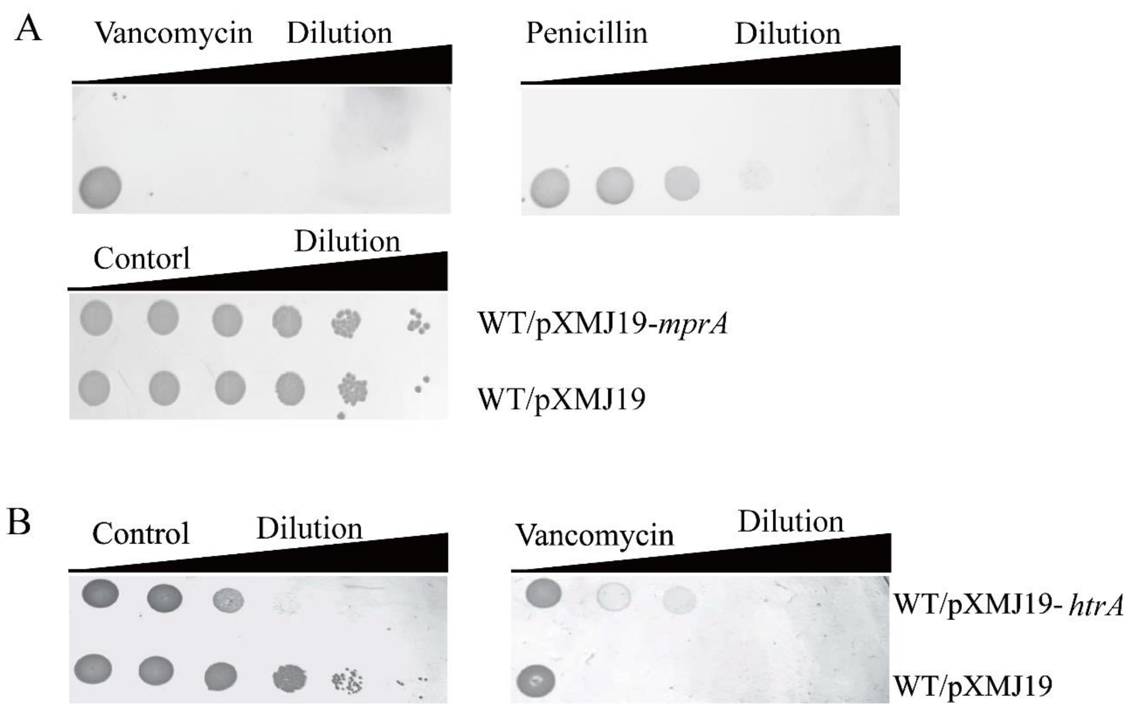


**Figure S4 Antibiotics sensitivity of *C. glutamicum* wide-type and overexpressing strains. Ten-fold serial dilutions of the *C. glutamicum* strains were prepared and 2 µL of each dilution was spotted on LBB agar plates containing 10 µg/mL chloramphenicol,** (A) 0.3 µg/mL vancomycin and 0.2 U/mL penicillin or (B) 0.3 µg/mL vancomycin.
